# Cancer-associated fibroblast-derived Gremlin 1 promotes breast cancer progression

**DOI:** 10.1186/s13058-019-1194-0

**Published:** 2019-09-18

**Authors:** Jiang Ren, Marcel Smid, Josephine Iaria, Daniela C. F. Salvatori, Hans van Dam, Hong Jian Zhu, John W. M. Martens, Peter ten Dijke

**Affiliations:** 1 Department of Cell and Chemical Biology, Oncode Institute, Leiden University Medical Center, Einthovenweg 20, 2333 ZC Leiden, The Netherlands; 2 Department of Medical Oncology, Erasmus MC Cancer Institute, Erasmus University Medical Center, Rotterdam, The Netherlands; 3 Department of Surgery, The Royal Melbourne Hospital, The University of Melbourne, Parkville, Australia; 4 Central Laboratory Animal Facility, Leiden University Medical Center, Leiden, the Netherlands

**Keywords:** Gremlin 1, Cancer-associated fibroblasts, Breast cancer, Invasion, Zebrafish

## Abstract

**Background:**

Bone morphogenetic proteins (BMPs) have been reported to maintain epithelial integrity and to antagonize the transforming growth factor β (TGFβ)-induced epithelial to mesenchymal transition. The expression of soluble BMP antagonists is dysregulated in cancers and interrupts proper BMP signaling in breast cancer.

**Methods:**

In this study, we mined the prognostic role of BMP antagonists *GREMLIN 1* (*GREM1*) in primary breast cancer tissues using in-house and publicly available datasets. We determined which cells express *GREM1* RNA using in situ hybridization (ISH) on a breast cancer tissue microarray. The effects of Grem1 on the properties of breast cancer cells were assessed by measuring the mesenchymal/stem cell marker expression and functional cell-based assays for stemness and invasion. The role of Grem1 in breast cancer-associated fibroblast (CAF) activation was measured by analyzing the expression of fibroblast markers, phalloidin staining, and collagen contraction assays. The role of Grem1 in CAF-induced breast cancer cell intravasation and extravasation was studied by utilizing xenograft zebrafish breast cancer (co-) injection models.

**Results:**

Expression analysis of clinical breast cancer datasets revealed that high expression of *GREM1* in breast cancer stroma is correlated with a poor prognosis regardless of the molecular subtype. The large majority of human breast cancer cell lines did not express *GREM1* in vitro, but breast CAFs did express *GREM1* both in vitro and in vivo. Transforming growth factor β (TGFβ) secreted by breast cancer cells, and also inflammatory cytokines, stimulated *GREM1* expression in CAFs. Grem1 abrogated bone morphogenetic protein (BMP)/SMAD signaling in breast cancer cells and promoted their mesenchymal phenotype, stemness, and invasion. Moreover, Grem1 production by CAFs strongly promoted the fibrogenic activation of CAFs and promoted breast cancer cell intravasation and extravasation in co-injection xenograft zebrafish models.

**Conclusions:**

Our results demonstrated that Grem1 is a pivotal factor in the reciprocal interplay between breast cancer cells and CAFs, which promotes cancer cell invasion. Targeting Grem1 could be beneficial in the treatment of breast cancer patients with high Grem1 expression.

**Electronic supplementary material:**

The online version of this article (10.1186/s13058-019-1194-0) contains supplementary material, which is available to authorized users.

## Background

Although carcinomas, which account for approximately 90% of human cancers, are derived from the epithelia, the tumor stroma exerts a powerful influence on cancer behavior, such as tumor cell growth, invasion, metastasis, and evading immune responses. The tumor stroma consists of cancer-associated fibroblasts (CAFs); vascular, inflammatory, and immune cells; and extracellular matrix (ECM) residing within or in the vicinity of a tumor [1]. CAFs are differentiated from quiescent fibroblasts and are associated with increased expression of myofibroblastic markers, such as vimentin, α-smooth muscle actin (αSMA), fibroblast activation protein (FAP), and fibroblast-specific protein 1 (FSP1, also known as S100A4) [2]. Tumors, including those from the breast, often display desmoplasia (a fibrillar network) that is mainly caused by CAFs, in that they produce and remodel ECM components, including collagen, fibronectin, and laminin [3]. The increased stiffness and abnormal physical structure of the ECM can promote tumor cell growth and metastatic dissemination and are also critical for the generation and maintenance of the CAF phenotype [3].

Bone morphogenetic proteins (BMPs) are secreted growth factors that belong to the transforming growth factor β (TGFβ) family [4]. Signaling by BMPs is initiated by binding their cognate transmembrane serine/threonine kinase receptors, which triggers the phosphorylation of intracellular SMAD1/5/8 (R-SMADs). Activated R-SMADs can form heteromeric complexes with SMAD4 that accumulate in the nucleus, where they can regulate transcriptional responses in concert with other DNA-binding transcription factors [4]. BMP signaling can elicit diverse and complex biological processes in development and disease, including cancer [5]. Many secreted BMP antagonists, which sequester BMP ligands and prevent their binding to receptors, have been identified [6]. Accumulating evidence indicates that several cancer types show dysregulated BMP signaling caused by a disequilibrium of BMPs and their antagonists. For example, BMP antagonists such as Noggin, Follistatin, and Chordin like (Chrdl)1 have been linked to induce osteoclast differentiation and promote osteolytic bone metastases [7, 8]. The BMP antagonist Coco permits a few dormant breast cancer cells to escape the quiescent state imposed by BMP signaling and thereby establish metastases [9].

Gremlin (Grem) 1 is a highly conserved glycoprotein belonging to the Cerberus and Dan subfamily of secreted BMP antagonists [10]. It preferentially interacts with BMP2, 4, and 7 [11]. Grem1 is the major BMP antagonist that maintains proper outgrowth and patterning during vertebral limb development [12]. Grem1 expression is also essential for cellular proliferation and branching morphogenesis in lung development and in kidney organogenesis [12, 13]. Aberrant expression in adults is associated with orofacial clefting [14], osteoarthritis [15], spontaneous bone fractures [16], and liver [17], lung [18], and renal [19] fibrosis. Grem1-mediated proangiogenic and proinflammatory activity appears to be independent of its effects on BMP [20, 21].

In several cancers, Grem1 reduces the negative effect of BMPs on stemness, proliferation, migration, and invasion of cancer cells [22–24]. In hereditary mixed polyposis syndrome, *GREM1* is predominantly expressed in the epithelium of the large bowel, where it disrupts homeostatic intestinal morphogen gradients and initiates colonic tumorigenesis [25, 26]. *GREM1* was also detected at the colorectal cancer desmoplastic invasion front, highlighting a potential role in cancer metastasis [27]. High levels of *GREM1* gene expression were observed in the stromal fibroblasts of many types of cancer [23, 28, 29], suggesting that CAFs are a potential source of Grem1. However, the effects of Grem1 on CAFs’ function and on the interaction between (breast) cancer cells and fibroblasts are unclear.

The results presented here support the idea that Grem1 is a clinical predictor of a poor prognosis in breast cancer. Mechanistically, Grem1 produced by CAFs promoted fibroblast activation in an autocrine manner and stimulated breast cancer cell stemness and invasion in a paracrine manner. Grem1 could be an attractive therapeutic target to interfere with breast cancer progression.

## Methods

### Data mining of gene expression in clinical patient samples and 52 breast cancer cell lines

In-house and publicly available gene expression datasets GSE2034 [30], GSE5327 [31], GSE2990 [32], GSE7390 [33], and GSE11121 [34] were used for *GREM1* (and *transforming growth factor β1/2/3* (*TGFB1/2/3*), *interleukin 1β* (*IL1B*), and *tumor necrosis factor α* (*TNFA*)) expression in lymph node-negative, non-(neo-)adjuvant-treated primary breast cancer patients with available metastasis-free survival data, leading to a cohort of 867 patients. Using the GSE41313 dataset [35], *GREM1*, *BMP*, and *BMP receptor* expression was assessed in silico in 52 breast cancer cell lines. Breast cancer dataset GSE14548 [28] was investigated to explore the *GREM1* expression in breast epithelium and stroma; this dataset was obtained using tissues from normal breast; grade I, II, and III ductal carcinoma in situ (DCIS), and invasive breast cancer tissues that were micro-dissected using a laser capture technique. In addition, the colorectal cancer dataset GSE39396 [36] was analyzed for *GREM1* expression; epithelial cells, leukocytes, fibroblasts, and endothelial cells were isolated by flow cytometry. Data were gathered from Gene Expression Omnibus (http://www.ncbi.nlm.nih.gov/geo/). Raw.cel files were processed using frozen robust multiarray analysis (fRMA) parameters (median polish) [37], after which the batch effects were corrected using ComBat [38].

### *GREM1* RNA in situ hybridization (ISH)

A matched breast cancer, adjacent (adenosis or hyperplasia, and cancer free) and adjacent normal tissue microarray (TMA) was purchased from US Biomax (BR724). *GREM1* RNA in situ hybridization was conducted with an RNAscope *GREM1* Probe (312831-C2, Advanced Cell Diagnostics) and a 2.5 HD Detection Kit – BROWN (322300, Advanced Cell Diagnostics). All procedures were performed by strictly following the manufacturer’s instructions. The ISH results were scanned by a digital slide scanner (Pannoramic 250 Flash III, 3DHISTECH). The presence of intracellular brown punctate dots was considered as positive staining. The signal intensity was scored utilizing a 5-point system: 0, no signals visible; 1, weak signals barely visible; 2, visible signals but not intensive; 3, moderate intensive signals; and 4, intensive signals. Scoring was evaluated independently by two observers with similar outcomes.

### Cell culture

The human breast cancer cell lines MDA-MB-231 and MCF7 were purchased from ATCC. The human human telomerase reverse transcriptase (hTERT)-immortalized breast CAFs 19TT cells have been previously described [39]. Human foreskin fibroblasts were obtained from Arti A. Ramkisoensing and have been previously published [40]. These cell lines and human embryonic kidney (HEK)293T cells were cultured in Dulbecco’s modified Eagle’s medium (DMEM, 11965092, Thermo Fisher Scientific) supplemented with 10% fetal bovine serum (FBS, 16000044, Thermo Fisher Scientific) and 100 U/ml penicillin/streptomycin (Pen/Strep, 15140148, Thermo Fisher Scientific). MCF10A (M1) human breast epithelial cell line and MCF10A-derived cell line MCF10A-Ras (M2) were generously provided by Dr. Fred Miller (Barbara Ann Karmanos Cancer Institute, Detroit, USA); both cell lines were cultured in DMEM/F12 medium (11039047, Thermo Fisher Scientific) supplemented with 5% horse serum (26050088, Thermo Fisher Scientific), 20 ng/ml epidermal growth factor (EGF, 01-107, Merck Millipore), 10 mg/ml insulin (91077C, Sigma-Aldrich), 100 ng/ml cholera enterotoxin (C8052, Sigma-Aldrich), 0.5 mg/ml hydrocortisone (H0135, Sigma-Aldrich), and 100 U/ml Pen/Strep. Human mesenchymal (HM), W18, and W21 fetal mesenchymal stem cells (MSCs) were isolated and previously described [40] and cultured in Minimum Essential Medium (MEM) α (32561037, Thermo Fisher Scientific) with 10% FBS, 100 U/ml Pen/Strep. All cell lines were maintained at 37 °C, 5% CO_2_, humidified incubator. All fibroblasts and MSCs were routinely cultured in 0.2% gelatin-coated (G9136, Sigma-Aldrich) flasks or plates during the whole experiment period to avoid possible activation caused by physical rigidity. All cell lines were monthly tested to verify the absence of mycoplasma, and human cell lines were authenticated by single nucleotide polymorphism (SNP) analysis.

### Plasmids, lentiviral transduction, and generation of stable cell lines

The human *GREM1* complementary DNA (cDNA) was cloned from cDNA by PCR and inserted into the pCDH lentiviral vector. pLV-mCherry has been described by our laboratory before [41]. pUltra-Smurf (blue fluorescent protein AmCyan) was obtained from Addgene (48974, Addgene). Human *GREM1* lentiviral shRNAs were obtained from the Sigma MISSION shRNA library. Five shRNAs were tested, and the two most effective shRNAs TRCN0000063833 (sh#1) and TRCN0000063837 (sh#2) were used.

Lentiviruses were produced by co-transfecting cDNA expression plasmids or shRNAs with helper plasmids pCMV-VSVG, pMDLg-RRE (gag/pol), and pRSV-REV into HEK293T cells using polyethyleneimine (PEI). Cell supernatants were harvested 48 h after transfection and stored at − 80 °C. MDA-MB-231 and MCF7 cells were labeled with mCherry by infecting for 24 h with mCherry-expressing lentiviral supernatants diluted 1:1 with normal culture medium in the presence of 5 ng/ml of polybrene (107689, Sigma-Aldrich). Forty-eight hours after infection, cells were placed under neomycin (A1720, Sigma-Aldrich) selection. 19TT and W21 cells were labeled with AmCyan and subjected to positive fluorescence-activated cell sorting (FACs). To obtain *GREM1* stable expressing cell lines, M1, M2, MDA-MB-231, and W21 cells were infected and selected with puromycin (P9620, Sigma-Aldrich). Puromycin was used at 1 μg/ml to maintain selection pressure. After infection with *GREM1* targeting shRNAs, 19TT cells were used within short term as 19TT cells are puromycin resistant already.

### Stimulation with conditioned medium (CM) or cytokines

MDA-MB-231 and MCF7 cells were grown to 70–80% confluency, washed two times with PBS, and incubated in serum-free DMEM for 24 h. Conditioned medium (CM) was then collected and passed through a 0.45-mm syringe filter (SLHP033RB, Merck Millipore).

19TT cells were treated with CM, recombinant human TGFβ3 (5 ng/ml, 8420-B3, R&D SYSTEMS and Andrew P. Hinck, University of Pittsburg, USA), interleukin 1β (IL1β, 10 ng/ml, 201-LB, R&D SYSTEMS), or tumor necrosis factor α (TNFα, 10 ng/ml, 210-TA, R&D SYSTEMS) for 1, 3, 6, 12, and 24 h. Buffer-treated controls were used in parallel. For antibody neutralization assays, TGFβ3 or CM was incubated with control (13C4) or TGFβ-neutralizing (1D11) antibody (generously provided by Sanofi Genzyme, Inc.) for 30 min (min) before treatment.

For inhibition of BMP signaling by recombinant human Grem1 (rhGrem1, 5190-GR, R&D SYSTEMS), rhGrem1 was pre-incubated with recombinant human BMP2/6 (5 ng/ml, 355-BM/507-BP, R&D SYSTEMS) for 30 min.

### Quantitative real-time PCR (qRT-PCR)

Total RNAs were isolated using the NucleoSpin RNA II kit (740955, BIOKE´). A total of 1 μg of RNA was reverse transcribed using the RevertAid First Strand cDNA Synthesis Kit (K1621, Thermo Fisher Scientific). Real-time PCR was conducted with GoTaq qPCR Master Mix (A6001, Promega) using CFX Connect Detection System (1855201, Bio-Rad). All target gene expression levels were normalized to *GAPDH*. The sequences of primers used to detect target human genes in qRT-PCR were listed in Additional file 1 Table S1.

### CAGA-luciferase reporter assay

HEK293T cells were seeded on a 24-well plate at approximately 5 × 10^4^ cells per well. The next day, cells in each well were co-transfected with 0.1 μg TGFβ/SMAD-inducible (CAGA)_12_ luciferase transcriptional reporter construct [42] and 0.08 μg β-galactosidase expression construct using PEI. After overnight incubation, cells were starved with serum-free medium. Eight hours later, serum-free media were removed and replaced by CM from breast cancer cell lines. One nanogram per milliliter TGFβ3 treatment was performed as a standard. After another overnight incubation, luciferase and β-galactosidase activities were measured. The luciferase activity was normalized based on the β-galactosidase activity.

### Western blotting

Cells were lysed with RIPA buffer containing 1 × complete protease inhibitor cocktail (11836153001, Roche). Protein concentrations were determined using a bicinchoninic acid protein assay kit (5000111, Bio-Rad). Proteins were separated by sodium dodecyl sulfate polyacrylamide gel electrophoresis (SDS-PAGE) and transferred onto 45-μm polyvinylidene difluoride (PVDF) membrane (IPVH00010, Merck Millipore). Membranes were blocked using 5% non-fat dry milk in Tris-buffered saline with 0.1% Tween 20 (655204, Merck Millipore) and probed with the respective primary and secondary antibodies. The signal was detected using Clarity™ Western ECL Substrate (1705060, Bio-Rad) and ChemiDoc Imaging System (17001402, Bio-Rad). The antibodies used for immunoblotting were raised against the following proteins: phospho-SMAD1/5/8 (pSMAD1/5/8, home-made) [43], αSMA (A2547, Sigma-Aldrich), fibronectin (F7387, Sigma-Aldrich), FAP (WH0002191M1, Sigma-Aldrich), collagen I (ab34710, Abcam), vimentin (5741, Cell signaling), and glyceraldehyde 3-phosphate dehydrogenase (GAPDH, MAB374, Merck Millipore). GAPDH was used as a protein loading control.

### Flow cytometry

Adherent cells were trypsinized and washed twice with 1% bovine serum albumin (BSA) (A2058, Sigma-Aldrich) in phosphate-buffered saline (PBS). The cells were then incubated with fluorescein isothiocyanate (FITC)-conjugated anti-human CD44 (347943, BD Biosciences) and R-phycoerythrin (PE)-conjugated anti-human CD24 (555428, BD Biosciences) antibodies (1:400 dilution) for 30 min at 37 °C in the dark. Fluorescein isothiocyanate (FITC)/PE-conjugated IgG isotypes (560952/560951, BD Biosciences) were used as a control. Cells were washed twice with 1% BSA in PBS and resuspended in 500 ml of PBS prior to analysis on a FACS Canto flow cytometer (BD Biosciences).

### Phalloidin staining

Cells were fixed in 4% formalin, permeabilized with 0.1% Triton X-100, and blocked with 5% BSA (A2058, Sigma-Aldrich) in PBS for 30 min. Then cells were stained with Alexa Fluor 488 Phalloidin (A12379, Thermo Fisher Scientific) to visualize filamentous (F)-actin. The nuclei were stained with 4′,6-diamidino-2-fenylindool (DAPI, 62248, Thermo Fisher Scientific). Images were taken by confocal microscopy (SP8, Leica Microsystems).

### Mammosphere formation assays

Single-cell suspensions of M1 cells were prepared in DMEM/F12 medium containing 1× B27 (17504044, Thermo Fisher Scientific), 20 ng/ml epidermal growth factor (01-107, Merck Millipore), 20 ng/ml fibroblast growth factors (PHG6015, Thermo Fisher Scientific), and 4 mg/ml heparin (H3149, Sigma-Aldrich). Then, 2000 cells/well were seeded into ultralow attachment 24-well plate (CLS3473-24EA, Corning). After 10 days of standard incubation, the numbers of spheres (> 75 mm diameter) were counted using an inverted microscope (DMi8, Leica Microsystems). For secondary sphere formation, primary spheres were dissociated with Accutase (A1110501, Thermo Fisher Scientific), followed by 25-gauge needles (Z192406, BD Biosciences) mechanically. Next, 2000 cells/well were replated. Sphere-forming efficiency was calculated as the number of spheres (average diameter = 100 μm) formed divided by the number of single cells originally seeded.

### Collagen gel contraction assays

The contraction assay [44] was performed to evaluate the contractility of 19TT cells with *GREM1* knockdown or *GREM1-*overexpressing W21 cells. Collagen gels were prepared by mixing fibroblast cell suspensions in serum-free medium and type I collagen (Corning, 354249) solution. The final cell density was 2.0 × 10^5^ cells/ml with 1 mg/ml collagen. A 0.5-ml mixture was casted into each well of a 24-well plate and allowed to polymerize for 30 min at 37 °C. Following gelatinization, another 0.5 ml of serum-free DMEM was added to the gel. Changes of gels were recorded by using a ChemiDoc Imaging System (17001402, Bio-Rad) at a fixed distance above the gels at 24, 48, and 72 h. The surface area of the gels was quantified by ImageJ software. The percentage of contraction was calculated using the formula 100% × (well surface area − gel surface area)/well surface area.

### Three-dimensional spheroid invasion assay

mCherry-labeled MDA-MB-231 or MCF7 cells and co-culture (1:1 mixture) with W21 or 19TT groups were prepared at 1000 cells/ml in complete DMEM. Drops of the single-cell suspension (30 μl) were placed onto the lids of 10-cm dishes, which were inverted over dishes containing 10 ml PBS. Hanging drop cultures were incubated 7 days allowing sufficient sedimentation and formation of one spheroid per drop. Images were taken by an inverted fluorescent microscope (DMi8, Leica Microsystems).

The 3D spheroid invasion assay was performed according to our previous study [45] with slight modifications. Single spheroids were embedded in the center of each well of a flat-bottom 96-well plate pre-coated with 50 μl of collagen mixture. Type I collagen (354249, Corning) was neutralized according to the manufacturer’s protocol. The collagen mixture was prepared by diluting neutralized collagen with serum-free medium to a final concentration of 2 mg/ml. Eight spheroids generated by each experimental setting were randomly chosen for embedding. After spheroid embedding, another 50 μl of collagen mixture was overlaid onto the collagen matrix in each well. The plate was incubated for 30 min at 37 °C to solidify the gels. Thereafter, 50 μl of serum-free medium was added to each well to prevent the surface from dehydrating. Plates were placed under standard cell culture conditions. Images were taken at days 0, 2, and 4 after embedding by using inverted fluorescence microscopy (DMi8, Leica Microsystems). The invasion was quantified by measuring the area occupied by cells using ImageJ software.

### Embryonic zebrafish intravasation and extravasation assay

Zebrafish xenograft breast cancer cell experiments were performed by injecting fluorescently labeled breast cancer cells into embryos at 48 h post-fertilization as described before [41]. Briefly, approximately 400 mCherry-labeled MDA-MB-231 cells were injected into the perivitelline space or the duct of Curvier (DoC) of transgenic zebrafish embryos (*fli*: *enhanced green fluorescent protein (EGFP)*), whose vasculature is marked in green. For co-injection, mCherry-labeled MDA-MB-231 cells and AmCyan-labeled W21 or 19TT cells were mixed at a ratio of 1:1. Then, approximately 400 mixed cells were injected into the zebrafish perivitelline space. Zebrafish embryos were maintained at 34 °C after injection, a compromise for both the fish and the human cell lines. Three days post-injection (dpi) into the perivitelline space, the MDA-MB-231 cells that intravasated from the cell mass toward the embryonic fish body within the head and tail regions were imaged and counted under a confocal microscope (SP5 STED, Leica Microsystems). At 5 dpi into the DoC, the number of MDA-MB-231 cells that extravasated individually from the circulation into the collagen fibers of the tail fin or the number of clusters formed by M2 cells collectively was analyzed. At least 200 zebrafish embryos were injected for each condition. After verification by microscopy, only correctly injected and viable zebrafish were used for experimental analysis. All experiments were repeated at least two times independently, and representative experiments are shown.

### Statistical analysis

Statistical analyses were performed using GraphPad Prism version 7.0. Numerical data from triplicates are presented as the mean ± standard deviation (s.d.), except for the analysis of zebrafish experiments, where a representative result is expressed as the mean ± standard error (s.e.m). Experiments were analyzed with an unpaired Student’s *t* test. *P* < 0.05 was considered statistically significant.

## Results

### High *GREM1* expression in breast tumors is associated with a poor prognosis

**Fig. 1:**
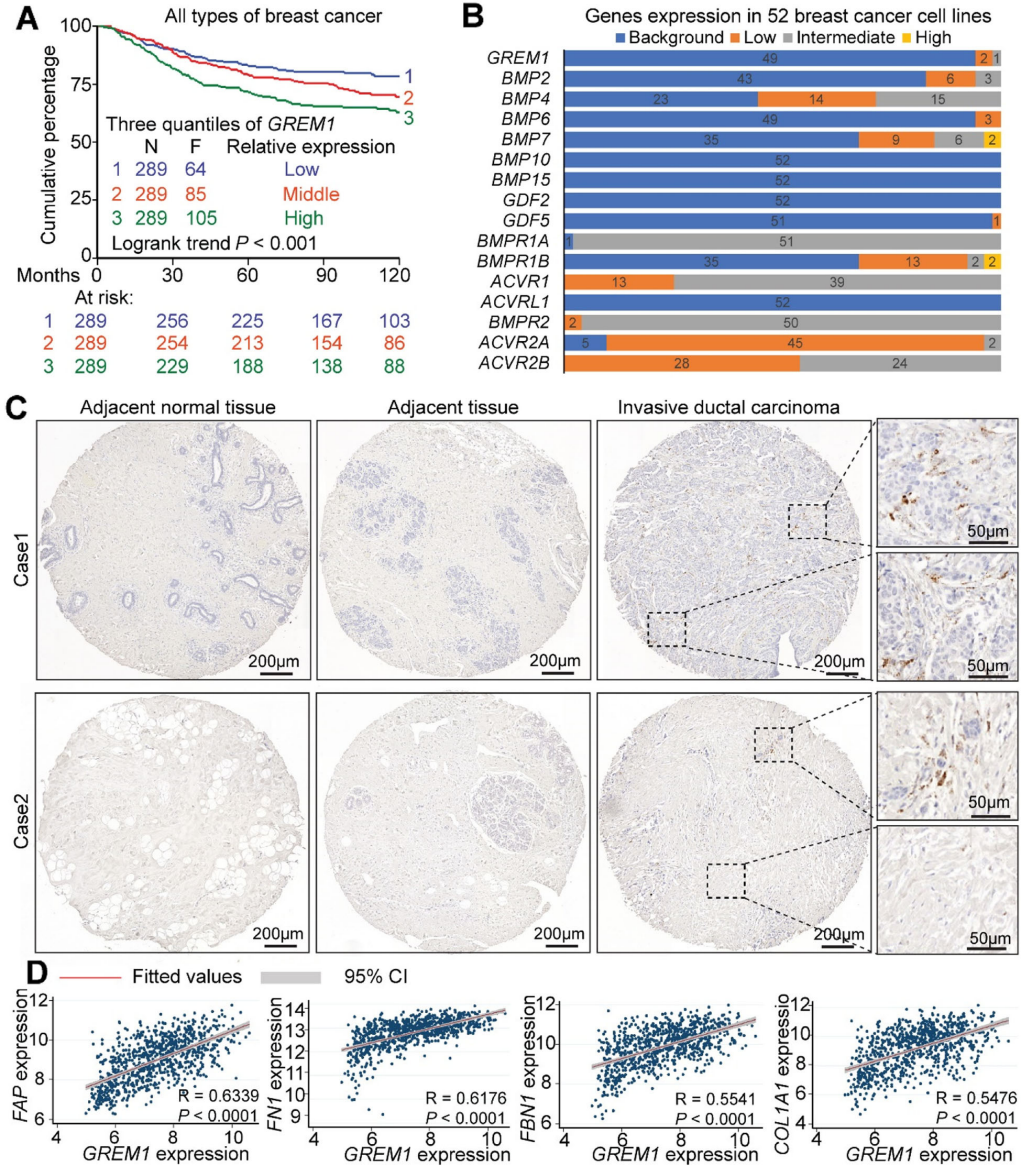
Stromal expression of *GREM1* predicts poor clinical outcome in breast cancer. **a** Kaplan-Meier survival curve in untreated lymph node-negative breast cancer patients. Based on *GREM1* mRNA expression (low, middle, and high), the subjects (*N* = 867) were divided into three quantiles. The endpoint is distant metastasis-free survival. **b** *GREM1*, *BMP*, and *BMP receptor* mRNA expression level in 52 breast cancer cell lines. The expression levels were categorized to four groups: background, low, intermediated, and high. **c** Human *GREM1* in situ hybridization shows restricted *GREM1* expression in fibroblast-like stromal cells surrounded by malignant breast epithelial cells. **d** Scatterplot showing positive correlation between the expression of *GREM1* and stromal genes/desmoplastic markers *FAP*, *FN1*, *FBN1*, and *COL1A1* in the clinical datasets. Pearson’s coefficient tests were performed to assess the statistical significance

BMPs have been reported to maintain epithelial integrity and to antagonize TGFβ-induced epithelial to mesenchymal transition (EMT), an important process for cancer cell invasion and metastasis [5]. Many soluble BMP antagonists have been described to be misexpressed and to interrupt proper BMP signaling in breast cancer [7–9]. We examined the prognostic role of soluble BMP antagonists in primary breast cancer using an in-house and publicly available cohort of 867 untreated lymph node-negative breast cancer patients (see the “Methods” section for datasets that were used). The median follow-up time of metastasis-free survival (MFS) was 94.1 months (range from 1 to 299.4 months). High expression of *GREM1* was found to be associated with a poor prognosis among all BMP antagonists that were examined. As shown in Fig. 1a, according to the *GREM1* mRNA expression level, the subjects were divided evenly into three quantiles: low, middle, and high. *GREM1* expression was inversely associated with MFS in this cohort, i.e., higher expression, and poorer outcome (low vs high: HR (hazard ratio) = 1.35, 95% confidence interval (CI) 1.15–1.57, log rank *P* = 0.00018; low vs middle: HR = 1.41, CI 1.02–1.96, *P* = 0.036; middle vs high: HR = 1.31, CI 0.98–1.74, *P* = 0.065). A similar association was observed when dividing subjects into two quantiles (Additional file 2: Figure S1a). Furthermore, high expression of *GREM1* correlated with a poor prognosis in all the breast cancer molecular subtypes examined: human EGF receptor (HER)2^+^, triple^−^, estrogen receptor (ER)^+^, and ER^−^ (Additional file 2: Figure S1b-e). Therefore, *GREM1* is a poor prognostic marker of metastasis-free survival in breast cancer regardless of the subtype.

### *GREM1* is expressed in cancer-associated fibroblasts

When we examined the *GREM1* expression in 52 human breast cancer cell lines by mining previously published datasets (see the “Methods” section), we found that only 3 breast cancer cell lines express low (MDA-MB-436 and HCC38) or intermediate (SUM149PT) levels of *GREM1*; all other 49 cell lines had no detectable expression (Fig. 1b). To explore the source of *GREM1* expression, we stained *GREM1* RNA by using in situ hybridization (ISH) in a breast cancer TMA, which comprised 24 matched cases of invasive ductal carcinoma, adjacent tissue, and adjacent normal tissue. As shown in Table 1 and Fig. 1c, we identified variable amounts of *GREM1* expressed in fibroblast-like cells, i.e., CAFs, whereas there were no detectable levels of *GREM1* in cancer-adjacent normal tissues or adjacent cancer-free breast tissues. None of the epithelial cells of breast cancers included in this study showed *GREM1*-positive expression. The *GREM1* expression in breast cancer tissue samples is thus mainly caused by the presence of tumor stroma. Moreover, using the in-house and publicly available primary breast cancer datasets, we observed a significant positive correlation between *GREM1* and markers for CAFs and tumor matrix stiffness/desmoplasia, such as *FAP*, *fibronectin* (*FN)1*, *fibrillin* (*FBN)1*, *collagen (COL)1A1*, *thrombospondin* (*THBS)2*, and *a-actin* (*ACTA)2* (Fig. 1d, Additional file 2: Figure S1f). Taken together, these results suggest that CAF-derived Grem1 might play a pivotal role in promoting breast tumor progression.

**Table 1:** RNA ISH scores for *GREM1* in matched breast cancer tissue microarray

RNA ISH score	Adjacent normal tissue (%)	Adjacent tissue (%)	Invasive ductal carcinoma (%)
0	24 (100)	24 (100)	4 (16.67)
1	0 (0)	0 (0)	6 (25.00)
2	0 (0)	0 (0)	5 (20.83)
3	0 (0)	0 (0)	5 (20.83)
4	0 (0)	0 (0)	4 (16.67)

### TGFβ secreted by cancer cells and inflammatory cytokines induces *GREM1* expression

**Fig. 2:**
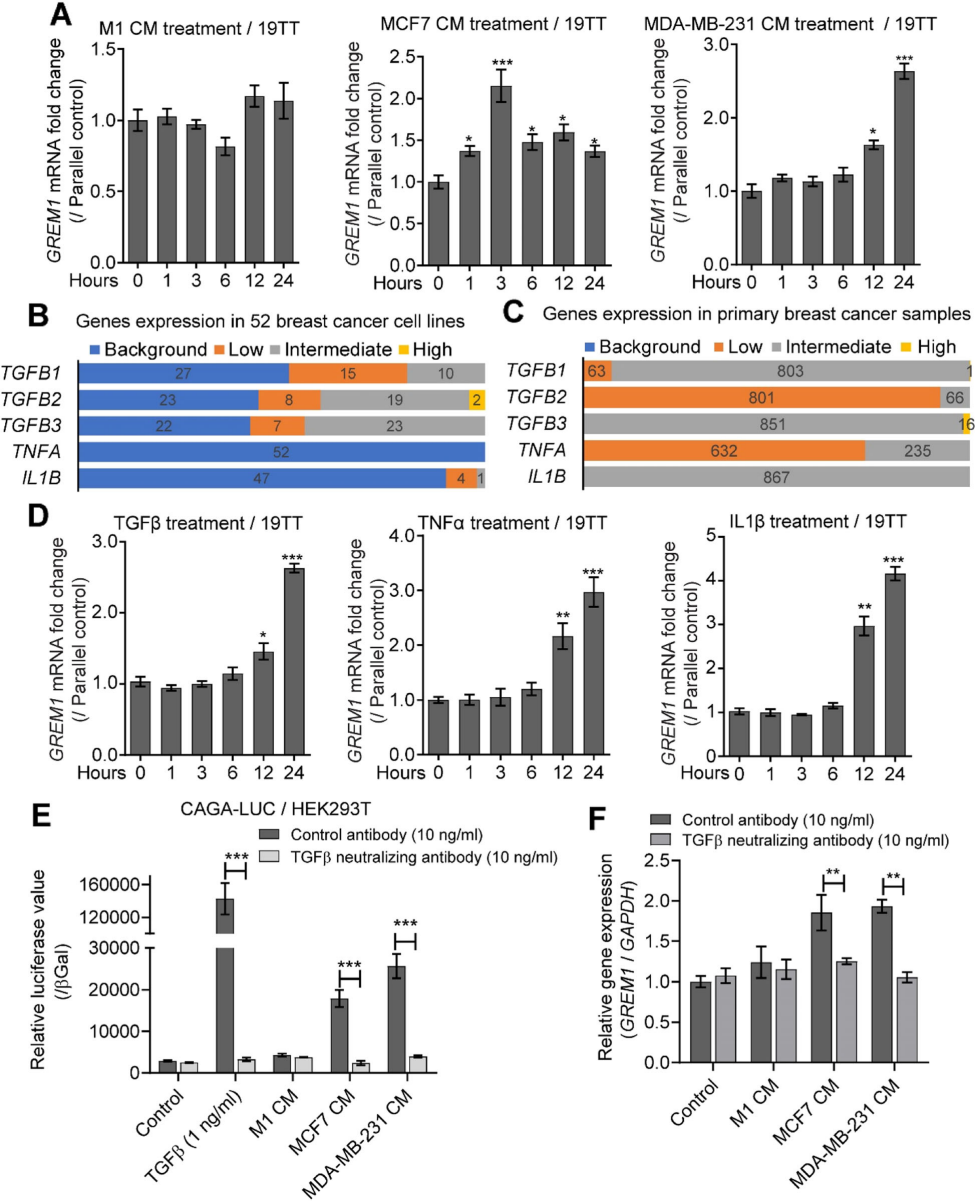
TGFβ secreted by breast cancer cells and inflammatory cytokines induce *GREM1* expression in CAFs. **a** *GREM1* expression in 19TT CAFs after treatment with conditioned medium (CM) from breast cell lines (M1, MDA-MB-21, or MCF7). The expression was normalized to the parallel time control of normal medium treatment. The results are expressed as the mean  ± s.d, *n* = 3. Student’s *t* test, *P < 0.05, ***P ≤ 0.001. **b** *TGFB1*, *TGFB2*, *TGFB3*, *TNFA*, and *IL1B* mRNA levels in 52 breast cancer cell lines. The expression levels were categorized into four groups: background, low, intermediated, and high. **c** *TGFB1/2/3*, *TNFA*, and *IL1B* expression in primary breast cancer samples. The expression level was categorized into four groups: background, low, intermediated, and high. **d** TGFβ3 (5 ng/ml), TNFα (10 ng/ml), or IL1β (10 ng/ml) induce *GREM1* expression in 19TT cancer-associated fibroblasts (CAFs). Expression was normalized to the parallel time control of buffer treatment. The results are expressed as the mean  ± s.d., *n* = 3. Student’s *t* test, *P < 0.05, **P ≤ 0.01, ***P ≤ 0.001. **e** Measurements of TGFβ activity in CM from breast cancer cell lines using a CAGA luciferase (LUC) reporter assay in HEK293T cells as a readout. TGFβ-neutralizing antibody (10 ng/ml) was added to demonstrate that luciferase activity in CM is due to the TGFβ activation and not activins or nodal. Recombinant TGFβ was added to control for the functionality of the assay. The value is normalized to β-galactosidase (βGal) activity. The results are expressed as the mean ± s.d, *n* = 3. Student’s *t* test, ***P ≤ 0.001. **f** The induction of *GREM1* expression in 19TT CAFs by CM from MCF7 and MDA-MB-21 is blocked by TGFβ-neutralizing antibody. The results are expressed as the mean ± s.d, *n* = 3. Student’s *t* test, **P ≤ 0.01

Analysis of GREM1 in tissue sections revealed that only the CAFs in close proximity to the cancer cells (tumor-stromal interface) showed high *GREM1* RNA expression (Fig. 1c, bottom panel). We therefore explored the possibility that fibroblasts express *GREM1* in response to factors secreted by cancer or inflammatory cells. We first collected CM from M1 immortalized normal breast cells and breast cancer cell lines MCF7 and MDA-MB-231. Treatment of 19TT CAFs (Fig. 2a) or W21 MSCs (Additional file 3: Figure S2a) with MDA-MB-231 or MCF7 cell CM resulted in a significant increase in *GREM1* mRNA levels. There was no effect of M1 CM on *GREM1* expression. To explore the factors that are responsible for inducing *GREM1* expression in CAFs, we analyzed by data mining the expression of *TGFB1/2/3* and inflammatory cytokines in breast cancer cell lines as well as in breast cancer tissues. We found that *TGFB1/2/3* are highly expressed in both breast cancer cell lines and tissues. Inflammatory cytokines, including *IL1B* and *TNFA*, were expressed in breast cancer tissues but only at very low levels in breast cancer cell lines (Fig. 2b, c). *IL1B* and *TNFA* expression in breast cancer tissues are thus likely caused by the stromal cells present in breast cancer tissue samples. Challenging 19TT CAFs (Fig. 2d) or W21 MSCs (Additional file 3: Figure S2b) with TGFβ3, TNFα, and IL1β promoted *GREM1* mRNA expression. Next, we analyzed whether TGFβ is secreted by cancer cells. MDA-MB-231 and MCF7, but not M1, were found to express active TGFβ (Fig. 2e). Importantly, the GREM1 expression-inducing activity of MDA-MB-231 or MCF7 cells could be blocked by a TGFβ-neutralizing antibody (Fig. 2f). Taken together, TGFβ secreted by cancer cells is the main determinant for inducing *GREM1* expression by CAFs. Within the tumor-stroma niche, inflammatory cells secreting cytokines may also contribute to *GREM1* expression by CAFs.

### Grem1 increases mammosphere formation

**Fig. 3:**
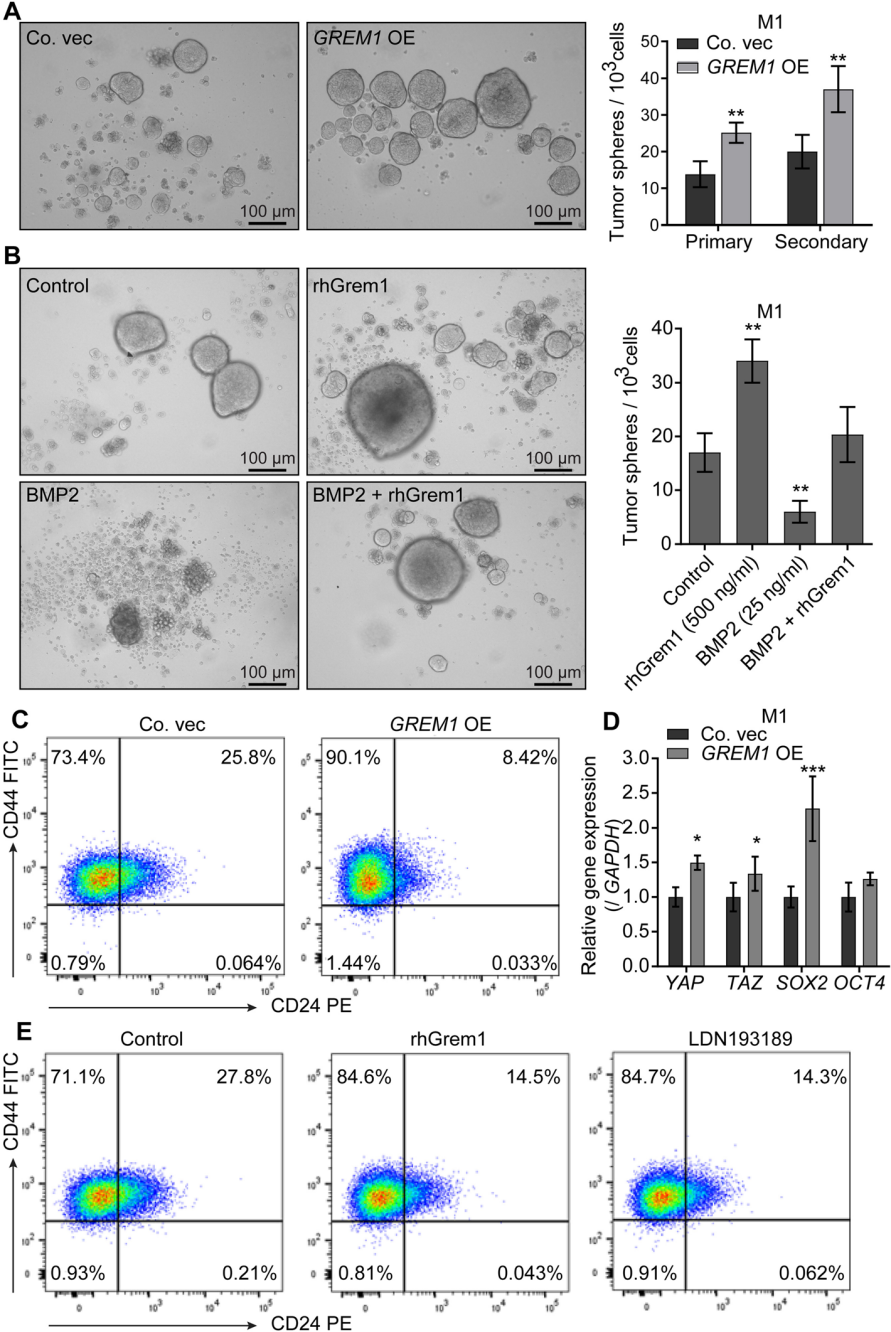
Grem1 maintains stemness in M1 cells. **a** *GREM1* overexpression (OE) induces more mammosphere formation in M1 cells. Left, representative images of mammospheres at 7 days. Right, the number of spheres formed per 1000 cells plated. The primary spheres were disintegrated and replated further. Secondary spheres formed were counted. The results are expressed as the mean ± s.d., *n* = 3. Student’s *t* test, **P ≤ 0.01. **b** Pro-mammosphere formation ability of recombinant human Grem1 (rhGrem1) protein (500 ng/ml) can be neutralized by BMP2 (50 ng/ml). Left, representative images of spheres at 7 days; right, the number of spheres formed per 1000 cells plated. The results are expressed as the mean ± s.d., *n* = 3. Student’s *t* test, **P ≤ 0.01. **c** Flow cytometry analysis shows that *GREM1* OE in M1 cells increases the stem population (CD44^+/high^ CD24^−/low^). **d** *GREM1* OE in M1 cells upregulates stem cell transcription factors. *GAPDH* was used as an internal control. The results are expressed as the mean ± s.d., *n* = 3. Student’s *t* test, *P < 0.05, ***P ≤ 0.001. **e** Flow cytometry analysis showing that 2 days of treatment with rhGrem1 (500 ng/ml) or the BMP type I receptor inhibitor LDN193198 (120 nM) also leads to an increase in the CD44^+/high^ CD24^−/low^ population

BMPs are reported to be inhibitors of cell stemness, suggesting that secreted Grem1 might oppositely affect the stem traits [9, 22, 46]. First, we confirmed that *BMPs* and *BMP receptors* are indeed expressed in breast cancer cell lines (Fig. 1b, Additional file 4: Figure S3a and b). Then, mammosphere formation assay was performed to assess the effect of Grem1 on mammary stem cell activity in vitro. *GREM1*-overexpressing M1 cells exhibited twofold more sphere formation compared to control cells in each of two subsequent passages (Fig. 3a). The administration of exogenous rhGrem1 showed a similar effect on mammosphere formation of M1 cells, whereas the administration of exogenous BMP2 mitigated sphere formation ability. The latter could be reversed by the concurrent administration of rhGrem1 (Fig. 3b). The surface expression of CD44^+/high^ CD24^−/low^ cells has been considered a stem population marker of breast cancers or cell lines [46]. Flow cytometry analysis demonstrated a significant increase in the CD44^+/high^ CD24^−/low^ cell subpopulation in *GREM1*-overexpressing M1 cells compared to the control (Fig. 3c). qRT-PCR revealed that *GREM1* OE (Fig. 3d) or rhGrem1 (Additional file 4: Figure S3c) increased the expression of transcriptional regulators *YAP*, *TAZ*, *SOX2*, and *OCT4*, which have been implicated in maintaining breast cancer stemness. Moreover, M1 cells treated with rhGrem1 or the BMP type I receptor inhibitor LDN193189 [47] also displayed more CD44^+/high^ CD24^−/low^ cells than non-treated control cells (Fig. 3e). These results suggest that Grem1 enhances the mammosphere formation of M1 cells by repressing BMP signaling.

### Grem1 promotes breast cancer cell invasion

**Fig. 4:**
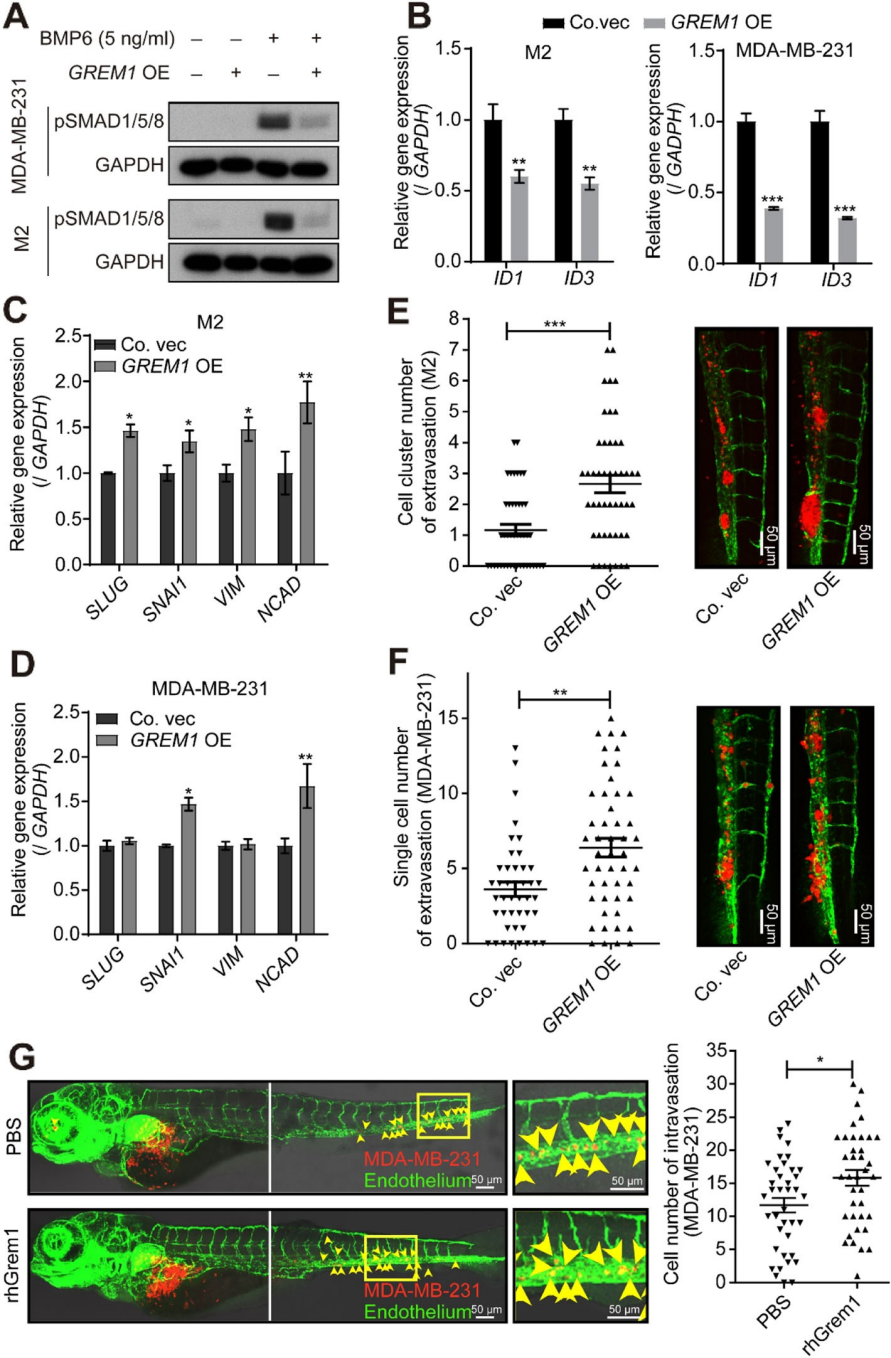
Ectopic expression of *GREM1* promotes cancer cell invasion in a zebrafish model. **a**, **b** *GREM1* overexpression (OE) inhibits BMP-induced SMAD1/5/8 phosphorylation (pSMAD1/5/8 (**a**)) and the BMP target genes *ID1* and *ID3* (**b**) in MDA-MB-231 and M2 cell lines. *GAPDH* was used as an internal control. The results are expressed as the mean ± s.d., *n* = 3. Student’s *t* test, **P ≤ 0.01. **c**, **d** *GREM1* OE upregulates the expression of EMT transcription factors and markers in M2 (**c**) and MDA-MB-231 (**d**) cells. *GAPDH* was used as an internal control. The results are expressed as the mean ± s.d., *n* = 3. Student’s *t* test, **P ≤ 0.01. **e**, **f** *GREM1* OE induces more clusters formation in M2 cells (**e**) and promotes the invasion of MDA-MB-231 cells (**f**) in zebrafish. Left, quantification of the number of extravasated cells/clusters at 5 days post-injection (dpi). Right, representative images: green, vasculature of zebrafish; red, mCherry-labeled cells. The results are expressed as the mean ± s.e.m., *n* = 2. Student’s *t* test, **P ≤ 0.01, ***P ≤ 0.001. **g** Perivitelline space injection of MDA-MB-231 cells supplemented with rhGrem1 (1 μg/ml) increases cell intravasation in zebrafish. Left, representative images: green, vasculature of zebrafish; red, mCherry-labeled cells. Right, quantification of the number of intravasated cells in each embryonic body at 3 days post-injection (dpi). The results are expressed as the mean ± s.e.m., *n* = 2. Student’s *t* test, *P < 0.05

To further characterize the role of Grem1 in breast cancer, we stably expressed Grem1 in the breast cancer cell lines M2 and MDA-MB-231 with a lentiviral vector. In a way, these transfected cell lines are somewhat reminiscent to the few breast cancer cell lines that express *GREM1*. In these *GREM1*-overexpressing cell lines, BMP-induced SMAD1/5/8 phosphorylation (Fig. 4a) and expression of BMP target genes ID1 and 3 (Fig. 4b) were clearly inhibited. Notably, the mRNA levels of the mesenchymal markers *SLUG*, *SNAI1*, *VIM*, and *NCAD* were increased by ectopic *GREM1* expression (Fig. 4c, d) or exogenous rhGrem1 treatment (Additional file 5: Figure S4b), suggesting that Grem1 induces a slightly more mesenchymal phenotype in these breast cancer cells. To test whether exposure to Grem1 also results in more invasive behavior, we introduced these cells into the blood circulation of embryonic zebrafish via DoC injection and examined extravasation 5 days post-injection (dpi) in the avascular tail fin area. Compared to the vector control, the *GREM1* overexpression group showed a higher number of extravasated M2 cell clusters (Fig. 4e) or MDA-MB-231 single cells (Fig. 4f). The BMP/SMAD signaling could be inhibited by exogenous administration of rhGrem1 (Additional file 5: Figure S4b). Next, we injected MDA-MB-231 cells suspended in PBS supplemented with or without rhGrem1 into the perivitelline space of embryonic zebrafish and examined the level of cells in circulation at 3 dpi. Exogeneous rhGrem1 increased cellular intravasation significantly, as more cells were found in the head and tail regions of zebrafish embryos (Fig. 4g).

### Grem1 promotes fibroblast activation

**Fig. 5:**
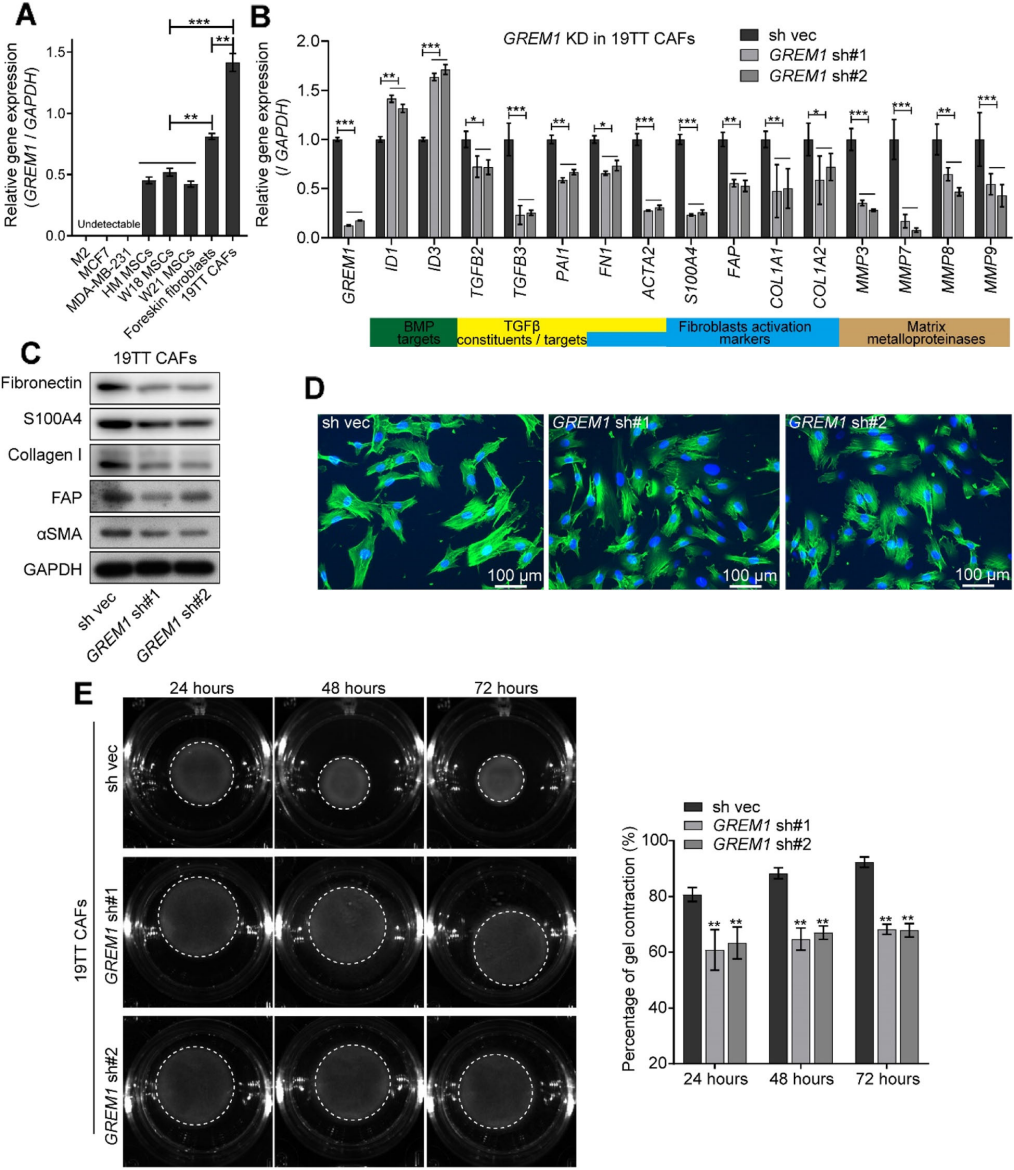
*GREM1* knockdown in 19TT breast cancer-associated fibroblasts (CAFs) attenuates fibrotic characteristics. **a** qRT-PCR comparison of relative *GREM1* expression in M2, MCF7, MDA-MB-231, HM, W18, and W21 fetal mesenchymal stem cells (MSCs), foreskin fibroblasts, and 19TT CAFs. *GAPDH* was used as an internal control. The results are expressed as the mean ± s.d., *n* = 3. Student’s *t* test, **P ≤ 0.01, ***P ≤ 0.001. **b** qRT-PCR analysis of the selected genes, BMP targets, TGFβ pathway constituents/targets, fibroblast activation markers, and matrix metalloproteinases in 19TT CAFs with/without shRNA-mediated *GREM1* knockdown. *GAPDH* was used as an internal control. The results are expressed as the mean ± s.d., *n* = 3. Student’s *t* test, *P ≤ 0.05, **P ≤ 0.01, ***P ≤ 0.001. **c** Western blot analysis to detect the changes in indicated proteins after *GREM1* knockdown in 19TT CAFs. **d** 19TT CAFs with/without *GREM1* knockdown were stained with fluorescein-phalloidin (green) to visualize F-actin. DAPI was used for nuclear staining (blue). **e** Collagen gel contraction assay. 19TT CAFs with/without *GREM1* knockdown were embedded in collagen gels. After 24, 48, and 72 h, the area of each gel (white dash circle) was imaged and quantified. Left, representative images of contracted gels. Right, the percentage of gel contraction. Quantification is shown in the “Methods” section. The results are expressed as the mean  ± s.d., *n* = 3. Student’s *t* test, **P ≤ 0.01

Grem1 is associated with fibrosis [17–19]. To explore the role of Grem1 in fibroblast activation, we first compared *GREM1* mRNA expression levels in foreskin fibroblasts, 19TT breast cancer CAFs, and HM, W18, and W21 human mesenchymal stem cells (MSCs). M2, MCF7, and MDA-MB-231 cancer cells served as a negative control. MSCs, which are considered fibroblast precursors and can differentiate into fibroblasts [2], showed the lowest expression of *GREM1*; *GREM1* expression in the foreskin fibroblasts, which represent normal fibroblasts, was significantly higher than that in MSCs, and 19TT CAFs showed the highest levels (Fig. 5a), indicating that *GREM1* expression increases during different stages of fibroblast activation. We next knocked down *GREM1* in 19TT CAFs. As shown in Fig. 5b, two shRNAs-mediated *GREM1* knockdown increased the mRNA expression of both *ID1* and *ID3* and decreased the expression of TGFβ signaling components and their target genes (*plasminogen activator inhibitor (PAI-1*), fibroblast activation markers, and *matrix metalloproteinases* (*MMPs*). *GREM1* knockdown in 19TT CAFs also led to decreased protein levels of FN1, S100A4, collagen I, FAP, and αSMA (Fig. 5c). This result suggests that Grem1 is a pivotal factor in fibroblast activation.

To examine whether Grem1 affects the cytoskeletal changes, we stained the cells with fluorescein-conjugated phalloidin to visualize filamentous (F)-actin. Indeed, *GREM1* knockdown in 19TT CAFs resulted in less prominent stress fibers and less organized bundles in the cytoplasm (Fig. 5d). More significantly, the ability of 19TT CAFs to contract collagen gels (a 3D model widely used for evaluating fibroblast-mediated matrix remodeling capacity) decreased significantly with *GREM1* knockdown (Fig. 5e). Moreover, *GREM1* overexpression in W21 MSCs (Additional file 6: Figure S5a) induced intensive myofibroblast-like characteristics (Additional file 5: Figure S5b-e). Consistent with this result, W21 MSCs treated with rhGrem1 or the selective BMP receptor kinase inhibitor LDN193189 exhibited an upregulation of genes which were inhibited by *GREM1* knockdown in 19TT CAFs (Additional file 6: Figure S5f). Overall, these observations imply that Grem1 is closely associated with the fibrogenic phenotype of breast CAFs.

### Fibroblast-derived Grem1 promotes breast cancer cell invasion in a 3D spheroid model

**Fig. 6:**
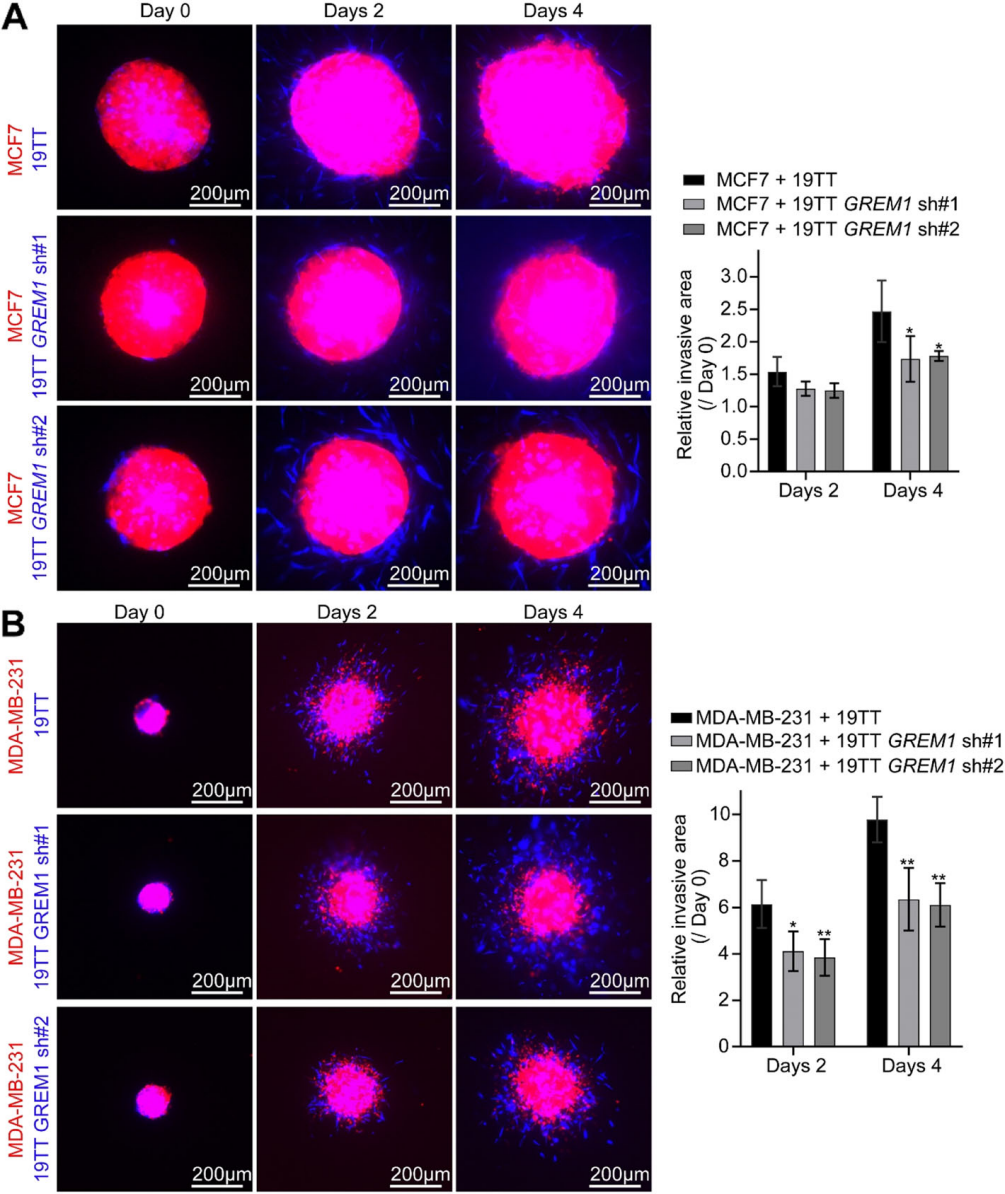
*GREM1* knockdown in 19TT breast cancer-associated fibroblasts (CAFs) impairs breast cancer cell invasion in a 3D spheroid invasion model. **a**, **b** Collagen invasion assay of co-culture spheroids. Eight spheroids per indicated group were embedded into collagen. Left, representative images of 3D spheroid invasion at days 0, 2, and 4. Red, MCF7 (**a**) or MDA-MB-231 (**b**) cells; blue, 19TT cells with/without *GREM1* knockdown. Right, relative invasion area was quantified as the area difference at days 2 and 4 relative to that at day 0. The results are expressed as the mean  ± s.d., *n* = 8. Student’s *t* test, *P < 0.05, **P ≤ 0.01

Previous studies have indicated that CAFs are propellants of cancer cell invasion [1, 2]. Prompted by the profibrotic role of Grem1, we further explored the roles of Grem1 in fibroblast-mediated cancer cell invasion using a 3D spheroid model. As illustrated in Additional file 7: Figure S6a, spheroids were produced from hanging drop co-cultures of mCherry-labeled breast cancer cells, MCF7 or MDA-MB-231 cells, and AmCyan-labeled 19TT CAFs. CAFs with or without *GREM1* knockdown were mixed with these breast cancer cells at a 1:1 ratio. The various resulting spheroids were embedded in collagen gel. As shown in Additional file 7: Figure S6b, the monocultured MCF7 spheroid showed a collective cell invasion phenotype in collagen, and in the presence of 19TT cells, the increased invasion of CAFs was measured at day 4. However, upon *GREM1* depletion in the 19TT CAFs, the co-culture spheroids showed strongly reduced invasion (Fig. 6a). Likewise, *GREM1* knockdown in the CAFs reduced the invasion of MDA-MB-231 cells in MDA-MB-231 and 19TT co-culture spheroids at days 2 and 4 (Fig. 6b).

### Fibroblast-derived Grem1 promotes breast cancer cell intravasation

**Fig. 7:**
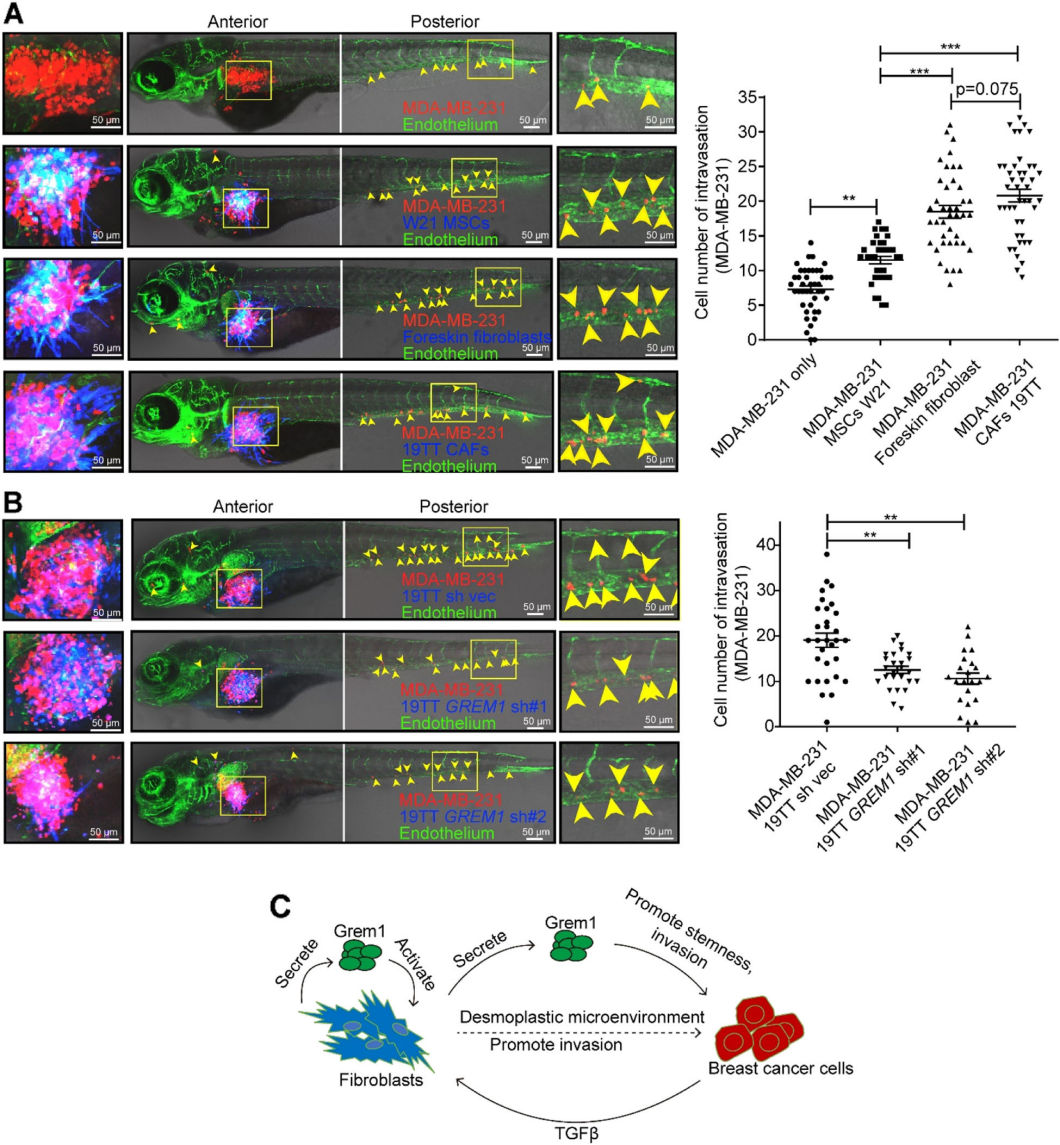
*GREM1* knockdown attenuates the ability of 19TT breast cancer-associated fibroblasts (CAFs) to promote breast cancer cell intravasation in a zebrafish co-injection model. **a** Perivitelline space single injection of MDA-MB-231 cells or co-injection of MDA-MB-231 cells and W21 mesenchymal stem cells (MSCs), foreskin fibroblasts, or 19TT CAFs, as indicated. The panel shows the representative images. Green, endothelium of zebrafish; red, mCherry-labeled MDA-MB-231; blue, converted from AmCyan-labeled MSCs or fibroblasts. Yellow arrowheads point to the single intravasated cells in the head and tail regions of zebrafish. Left, cell migration in the perivitelline space; middle, the image of a zebrafish embryo body. Right, visualization of the intravasated cells in the posterior of the embryo. The graph shows the quantification of the number of intravasated cells in each embryonic body at 3 days post-injection (dpi). The results are expressed as the mean ± s.e.m., *n* = 2. Student’s *t* test, **P ≤ 0.01, ***P ≤ 0.001. **b** Perivitelline space co-injection of MDA-MB-231 cells and 19TT CAFs with/without *GREM1* knockdown. The panel and graph description are the same as described in **a**. The results are expressed as the mean ± s.e.m., *n* = 2. Student’s *t* test, **P ≤ 0.01. **c** Schematic of the working model of Grem1 function in breast cancer progression. Grem1 expression in fibroblasts is induced by factors (such as TGFβ from breast cancer cells or maybe other stromal cells (that produce inflammatory cytokines). Grem1 could activate fibroblasts into CAFs. CAFs might present a desmoplastic microenvironment, thereby promote cancer cell invasion. Grem1 itself could promote the stemness and invasion of breast cancer cells

Next, we examined the role of fibroblast-expressed Grem1 in breast cancer cell invasion in vivo. We injected mCherry-labeled MDA-MB-231 cells into the perivitelline space of zebrafish in the absence or presence of either AmCyan-labeled W21 MSCs, foreskin fibroblasts, or 19TT CAFs. As depicted in Fig. 7a, intravasation of the MDA-MB-231 cells was significantly increased when co-implanted with W21, validating a previous study in which MSCs promoted cancer metastasis [48]. Importantly, this intravasation was much more enhanced by the foreskin fibroblasts and even more so by the 19TT CAFs, suggesting a correlation with their *GREM1* expression level. Indeed, the ectopic expression of *GREM1* in W21 cells resulted in enhanced MDA-MB-231 cell intravasation upon co-injection (Additional file 8: Figure S7). Consistent with this result, *GREM1* knockdown mitigated the promotion role of 19TT CAFs on MDA-MB-231 cell intravasation (Fig. 7b).

## Discussion

Our work has uncovered a strong association between high *GREM1* expression in breast tumor biopsies and a poor prognosis. We provide mechanistic insights into GREM1’s key role in facilitating breast cancer progression using in vitro and in vivo studies. Grem1 is highly expressed by CAFs at the invasion front; its expression can be promoted by factors, such as TGFβ released by breast cancer cells and inflammatory cytokines. Grem1 mediates the fibrogenic activation of CAFs in an autocrine manner. Grem1 has a direct effect on cancer cell invasion and stemness, evidenced by the fact that it promoted a slightly more mesenchymal/stemness phenotype in breast cancer cells. It could also contribute indirectly to this process via its potent effects on fibroblast activation. In this way, Grem1 promotes the formation of a microenvironment conducive to breast cancer cell invasion. Thus, Grem1 is a key determinant of the mutual interplay between breast cancer cells and CAFs (Fig. 7c).

Although we found an association between Grem1 and poor breast cancer prognosis, the prognostic significance of Grem1 in different cancer types is not consistent. For example, Grem1 expression correlates with progression-free survival in pancreatic neuroendocrine tumors [49] and colorectal cancer [50], but it is an indicator of poor progression-free survival in cervical cancer [51]. Grem1 may have different roles in different tumor types, but this may be dependent on the experimental setup, the analysis of expression in complete tumors vs stromal expression specifically, and/or the determining the levels of RNA vs protein. For instance, when testing commercial antibodies on tissue sections, including sections of *GREM1*-deficient animals, we found that the detected signals may not have been specific for Grem1 (data not shown). To avoid these putatively non-specific measurements, we determined *GREM1* mRNA levels by in situ hybridization.

The mRNA detection method revealed that *GREM1* was exclusively expressed by CAFs. Our findings are supported by our data mining of publicly available datasets. We analyzed breast cancer dataset GSE14548 generated by Ma and colleagues [28], which separated epithelial and stromal tissues, and in this dataset, *GREM1* was found mainly expressed in the (invasive) breast cancer stroma, and there was no *GREM1* expression observed in normal epithelium and stroma (Additional file 9: Figure. S8a). In addition, we mined a colon cancer dataset GSE39396 in which epithelial cells, leukocytes, fibroblasts, and endothelial cells were separately isolated by FACs and thereafter profiled. Consistent with our results, only fibroblasts were found to express GREM1 (Additional file 9: Figure S8b).

We found that *GREM1* expression in CAFs is particularly high in close vicinity of cancer cells. This is consistent with previous reports in which Grem1 was found to be highly expressed in CAFs in the microenvironment of basal cell carcinoma (and other tumors) compared to normal tissue counterparts [23] and a study of colorectal cancer, in which Grem1 was found to be expressed at the invasion fronts in CAFs and to mediate the loss of cancer cell differentiation [3]. We identified TGFβ secreted by cancer cells as a strong driver of *GREM1* expression by CAFs. Moreover, clinical breast cancer samples were also found to highly express *TGFB1/2/3* suggesting that these findings are of clinical relevance. Such invasion fronts are rich in inflammatory cells [52]. Consistent with this result, we found that inflammatory cytokines IL1β and TNFβ induced *GREM1* expression in CAFs. Moreover, *GREM1* expression correlated with mesenchymal marker expression in tumor samples. The latter observation indicates that Grem1 at the invasion front may contribute to the desmoplastic phenotype (Fig. 7).

We observed a striking activation of fibrogenesis in fibroblasts and in CAFs by Grem1. Depletion or ectopic expression of Grem1 in CAFs demonstrated that Grem1 expression is positively linked to the expression of TGFβ ligands and target genes, mesenchymal markers, extracellular matrix proteins, and matrix metalloproteinase (MMP) remodeling factors at the mRNA level, as well as with fibronectin, S100A4, collagen I, FAP, and αSMA at the protein level. In addition, Grem1 promoted actin stress fiber formation and collagen gel contraction. These expression patterns are a characteristic of a fibrogenic response and fibroblast activation. The Grem1-induced responses may be mediated by TGFβ pathway activation; TGFβ is a strong inducer of fibrogenesis and an activator of fibroblasts [53]. With TGFβ being a strong inducer of Grem1 and vice versa, it may act in a feed-forward loop.

Multiple studies have shown that CAFs create a microenvironment suitable for cancer cell invasion [1, 2], which we further demonstrated in this study in vivo by co-injection of breast cancer cells with fibroblasts/CAFs into the zebrafish perivitelline space. Thus, the profibrogenic ability of Grem1 could contribute to its role in promoting cancer cell invasion mediated by activated fibroblasts and CAFs. 3D co-culture of breast cancer cells with CAFs in collagen demonstrated that Grem1 is critical for invasion. In accordance with these results, Grem1 strongly promoted intravasation in a zebrafish co-injection xenograft model. Moreover, by injecting ectopic Grem1-producing M2 and MDA-MB-231 cells into the DoC of zebrafish embryos, we found that Grem1 strongly promoted the extravasation of cancer cells. These results may explain the clinical association between Grem1 expression in tumors and a poor prognosis of MFS.

Mechanistically, Grem1 exerts its effects by antagonizing selective BMPs [11]. Consistent with this notion, we found that BMP-induced SMAD1/5 phosphorylation is inhibited in breast cancer cells and in CAFs. In addition, depletion of endogenous Grem1 in CAFs upregulates BMP/SMAD-dependent *ID1*/*ID3* expression while the addition of rhGrem1 has the opposite effect. Moreover, treatment with a selective BMP receptor kinase inhibitor mimicked the effect of exogenous Grem1 protein by promoting mammosphere formation and fibrogenic marker expression. However, our results do not exclude the possibility that Grem1 also can act via BMP-independent pathways [24]. For example, induction of TGFβ expression by Grem1 may occur independently of BMP antagonism. Grem1 was found to promote cell viability, migration, and invasion in glioma [54] and the invasive phenotype of mesothelioma [55] by activating TGFβ/SMAD signaling. Moreover, Grem1 may promote breast tumorigenesis by acting on signaling pathways distinct from TGFβ family signaling; in renal tubular cells, Grem1 has been reported to signal via the vascular endothelial growth factor receptor 2 (VEGFR2) pathway [21] to promote angiogenesis [56] and to mediate inflammation and the infiltration of immune-inflammatory cells [57]. Furthermore, Grem1 may act directly or indirectly by sequestering BMP on endothelial cells and immune cells and thereby promote tumorigenesis. Irrespective of the precise mechanisms, our results demonstrate potent pro-tumorigenic effects of Grem1 on cancer cells and CAFs in vitro in mono- and in co-culture, as well as a key in vivo role for Grem1 in stimulating extravasation and for Grem1-producing CAFs in mediating the intravasation of breast cancer cells. These two processes, extravasation and intravasation, are the key steps in the dissemination and distant colonization of primary cancer cells.

## Conclusion

Our results identified Grem1 as a driving force of breast cancer progression by affecting the behavior of both cancer cells and neighboring CAFs. Antibodies that neutralize Grem1’s function in the Grem1-BMP interaction have been described, which may be beneficial not only for the treatment of pulmonary arterial hypertension [58] but also for breast cancer (by inhibiting breast cancer progression). In addition, BMP agonists that are engineered to prevent interactions with Grem1, as has been performed for Noggin [59], or BMP-mimetic small-molecule drugs [60, 61], could be beneficial in the treatment of breast cancer patients with high Grem1 expression.

## Additional files

Additional file 1: **Table S1.** Quantitative real-time PCR Primers. (XLSX 12 kb)

Additional file 2: **Figure S1.** Related to Fig. 1. a Kaplan-Meier analysis of metastasis free survival based on *GREM1* expression. Endpoint is distant metastasis free survival (MFS). b-e Kaplan-Meier survival analysis of different breast cancer molecular subtypes, HER2^+^ (b), Triple^-^ (c), ER^+^ (d), and ER^-^ (e). The subjects were divided into 3 quantiles. Endpoint is distant MFS. f Scatterplot showing the positive correlation between *GREM1* and stromal genes / desmoplastic markers expression in clinical datasets. Pearson’s coefficient tests were performed to assess statistical significance. (DOCX 766 kb)

Additional file 3: **Figure S2.** Related to Fig. 2. a *GREM1* expression in W21 MSCs after treatment with conditioned medium (CM) from breast cell lines (M1, MDA-MB-21, MCF7). Expression was normalized to the parallel time control of normal medium treatment. The results are expressed as the mean ± s.d, *n* = 3. Student’s t test, *P < 0.05, ***P ≤ 0.001. b TGFβ3 (5 ng/ml), or TNFα (10 ng/ml), or IL1β (10 ng/ml) induces *GREM1* expression in W21 mesenchymal stem cells (MSCs). Expression was normalized to the parallel time control of buffer treatment. The results are expressed as the mean ± s.d., *n* = 3. Student’s t test, *P < 0.05, **P ≤ 0.01, ***P ≤ 0.001. (DOCX 296 kb)

Additional file 4: **Figure S3.** Related to Fig. 3. a, b qRT-PCR measurement for BMPs and BMP receptors in M1, MDA-MB-231 and MCF7 cell lines. ∆Ct values are labeled to show expression abundance. **c** rhGrem1 upregulates stem cell transcription factors in M1 cells. *GAPDH* was used as an internal control. The results are expressed as the mean ± s.d., *n* = 3. Student’s t test, *P < 0.05, **P ≤ 0.01. (DOCX 368 kb)

Additional file 5: **Figure S4.** Related to Fig. 4. a *GREM1* OE upregulates the expression of EMT transcription factors and markers in M1 cells. *GAPDH* was used as an internal control. The results are expressed as the mean ± s.d., *n* = 3. Student’s t test, *P < 0.05, **P ≤ 0.01, ***P ≤ 0.001. b exogenous administration of rhGrem1 inhibits BMP-induced SMAD1/5/8 phosphorylation (pSMAD1/5/8) in MDA-MB-231 and M2 cell lines. (DOCX 175 kb)

Additional file 6: **Figure S5.** Related to Fig. 5. *GREM1* overexpression (OE) in fetal mesenchymal stem cells (MSCs) W21 shows fibroblast-like characteristics. a Stable *GREM1* OE in MSCs W21 inhibits BMP6 (5 ng/ml) induced SMAD1/5/8 phosphorylation (pSMAD1/5/8). Left, relative mRNA level determined by qRT-PCR. *GAPDH* was used as internal control. The results are expressed as the mean ± s.d., n = 3. Student’s t test, ***P ≤ 0.001. b qRT-PCR analysis of selected BMP targets, TGFβb pathway constituents/targets, fibroblast activation markers, matrix metalloproteinases, in W21 MSCs with/without *GREM1* stable OE. *GAPDH* was used as internal control. The results are expressed as the mean ± s.d., n = 3. Student’s t test, *P < 0.05, **P ≤ 0.01, ***P ≤ 0.001. c Western blot to detect indicated proteins level change after *GREM1* OE in W21 MSCs. d W21 MSCs with/without *GREM1* OE were stained with fluorescein-phalloidin (green) to visualize F-actin. DAPI was used for nuclear staining (blue). e Collagen gel contraction assay. W21 MSCs with/without *GREM1* OE were embedded in collagen gels. After 24, 48, and 72 h, the area of each gel (white dash circle) was imaged and quantified. Left, representative images of contracted gels. Right, percentage of gel contraction gel. Quantification is shown in Methods. The results are expressed as the mean  ± s.d., n = 3. Student’s t test, *P < 0.05, **P ≤ 0.01. f qRT-PCR analysis of selected genes in W21 MSCs after 48 hours treatment with recombinant human Grem1 (rhGrem1) protein (500 ng/ml) or BMP type I receptors inhibitor LDN193198 (120 nM). *GAPDH* was used as internal control. The results are expressed as the mean ± s.d., n = 3. Student’s t test, *P < 0.05, **P ≤ 0.01, ***P ≤ 0.001. (DOCX 447 kb)

Additional file 7: **Figure S6.** Related to Fig. 6. Spheroid invasion assays. **a** Schematic illustration of spheroid production. Briefly, mCherry-labeled MDA-MB-231 or MCF7 cells (Red) were mixed with AmCyan (converted to blue)-labeled 19TT breast cancer-associated fibroblasts (CAFs) at a ratio of 1:1. Mixtures were cultured for 7 days in hanging drops to obtain spheroids. **b** 19TT CAFs promotes MCF7 cells invasion. Left, representative images of spheroids at days 0, 2, and 4. Red, MCF7 cells; Blue, 19TT CAFs. Right, the relative invasion area was quantified as area difference at days 2 and 4, relative to day 0. The results are expressed as the as the mean  ± s.d., *n* = 8. Student’s t test, **P ≤ 0.01. (DOCX 197 kb)

Additional file 8: **Figure S7.** Related to Fig. 7. *GREM1* overexpression (OE) in W21 mesenchymal stem cells (MSCs) promotes breast cancer cells intravasation in zebrafish embryo perivitelline space coinjection model. Perivitelline space co-injection of MDA-MB-231 cells and W21 MSCs with/without *GREM1* stable OE. The panels show representative images. Green, endothelium of zebrafish; Red, mCherry-labelled MDA-MB-231; Blue, converted from AmCyan-labelled W21. Yellow arrowheads point to single intravasated cells in the head and tail regions of zebrafish. Left, cells migration in the perivitelline space; middle, image of zebrafish embryo body; Right, visualization of intravasated cells in the posterior of embryo. The graph shows quantification of the number of intravasated cells in each embryonic body at 3 days post injection (dpi). The results are expressed as the mean ± s.e.m., *n*=2. Student’s t test, **P ≤ 0.01. (DOCX 269 kb)

Additional file 9: **Figure S8.** Related to Fig. 1. a *GREM1* mRNA expression in epithelium and stroma compartments in breast cancer dataset GSE14548. Epithelium and stroma were extracted from normal breast, grade I, II, III ductal carcinoma in situ (DCIS) and invasive breast cancer tissue using laser capture. b *GREM1* expression in epithelial cells, leukocytes, fibroblasts and endothelial cells in colorectal cancer dataset GSE39396. The each specific type of cells were isolated by flow cytometry. (DOCX 177 kb)

## Data Availability

All remaining data and materials are available from the authors upon reasonable request.

## Abbreviations

ACTA: β-Actin
αSMA: α-Smooth muscle actin
BMPs: Bone morphogenetic proteins
BSA: Bovine serum albumin
CAFs: Cancer-associated fibroblasts
CM: Conditional medium
3D: Three-dimensional
cDNA: Complementary DNA
COL: Collagen
DCIS: Ductal carcinoma in situ
DoC: Duct of Curvier
DMEM: Dulbecco’s modified Eagle’s medium
dpi: Days post-injection
ECM: Extracellular matrix
EGFP: Enhanced green fluorescent protein
EMT: Epithelial to mesenchymal transition
ER: Estrogen receptor
FACs: Fluorescence-activated cell sorting
FAP: Fibroblast activation protein
FBS: Fetal bovine serum
FN: Fibronectin
FSP1: Fibroblast-specific protein 1
fRMA: Frozen robust multiarray analysis
GAPDH: Glyceraldehyde 3-phosphate dehydrogenase
HER: Human epidermal growth factor receptor
HR: Hazard ration
ID1: Inhibitor DNNA binding
IL1β: Interleukin 1β
ISH: In situ hybridization
Grem1: Gremlin 1
M1: MCF10A
M2: MCF10A-Ras
MEM: Minimum Essential Medium
MMP: Matrix metalloproteinase
MSC: Mesenchymal stem cells
MFS: Metastasis-free survival
PEI: Polyethylenimine
Pen/Strep: Penicillin/streptomycin
rh: Recombinant human
s.d.: Standard deviation
s.e.m.: Standard error
SNP: Single nucleotide polymorphism
TGFβ: Transforming growth factor β
TNFα: Tumor necrosis factor α
TMA: Tissue microarray
THBS: Thrombospondin
qRT-PCR: Quantitative real-time polymerase chain reaction

## References

[1] Madar S, Goldstein I, Rotter V (2013). ‘Cancer associated fibroblasts’–more than meets the eye. Trends Mol Med.

[2] Kalluri R (2016). The biology and function of fibroblasts in cancer. Nat Rev Cancer.

[3] Karagiannis GS, Poutahidis T, Erdman SE, Kirsch R, Riddell RH, Diamandis EP (2012). Cancer-associated fibroblasts drive the progression of metastasis through both paracrine and mechanical pressure on cancer tissue. Mol Cancer Res.

[4] Katagiri T, Watabe T (2016). Bone morphogenetic proteins. Cold Spring Harb Perspect Biol.

[5] Ren Jiang, ten Dijke Peter (2017). Bone Morphogenetic Proteins in the Initiation and Progression of Breast Cancer. Bone Morphogenetic Proteins: Systems Biology Regulators.

[6] Brazil DP, Church RH, Surae S, Godson C, Martin F (2015). BMP signalling: agony and antagony in the family. Trends Cell Biol.

[7] Tarragona M, Pavlovic M, Arnal-Estapé A, Urosevic J, Morales M, Guiu M, Planet E, González-Suárez E, Gomis RR (2012). Identification of NOG as a specific breast cancer bone metastasis-supporting gene. J Biol Chem.

[8] Mock K, Preca B, Brummer T, Brabletz S, Stemmler M, Brabletz T (2015). The EMT-activator ZEB1 induces bone metastasis associated genes including BMP-inhibitors. Oncotarget.

[9] Gao H, Chakraborty G, Lee-Lim AP, Mo Q, Decker M, Vonica A, Shen R, Brogi E, Brivanlou AH, Giancotti FG (2012). The BMP inhibitor Coco reactivates breast cancer cells at lung metastatic sites. Cell.

[10] Nolan K, Thompson TB (2014). The DAN family: modulators of TGFβ signaling and beyond. Protein Sci.

[11] Church RH, Krishnakumar A, Urbanek A, Geschwindner S, Meneely J, Bianchi A, Basta B, Monaghan S, Elliot C, Strömstedt M (2015). Gremlin1 preferentially binds to bone morphogenetic protein-2 (BMP-2) and BMP-4 over BMP-7. Biochem J.

[12] Khokha MK, Hsu D, Brunet LJ, Dionne MS, Harland RM (2003). Gremlin is the BMP antagonist required for maintenance of Shh and Fgf signals during limb patterning. Nat Genet.

[13] Michos O, Panman L, Vintersten K, Beier K, Zeller R, Zuniga A (2004). Gremlin-mediated BMP antagonism induces the epithelial-mesenchymal feedback signaling controlling metanephric kidney and limb organogenesis. Development.

[14] Mangold E, Ludwig KU, Birnbaum S, Baluardo C, Ferrian M, Herms S, Reutter H, de Assis NA, Al Chawa T, Mattheisen M (2010). Genome-wide association study identifies two susceptibility loci for nonsyndromic cleft lip with or without cleft palate. Nat Genet.

[15] Tardif G, Pelletier J-P, Boileau C, Martel-Pelletier J (2009). The BMP antagonists follistatin and gremlin in normal and early osteoarthritic cartilage: an immunohistochemical study. Osteoarthr Cartil.

[16] Gazzerro E, Pereira RC, Jorgetti V, Olson S, Economides AN, Canalis E (2005). Skeletal overexpression of gremlin impairs bone formation and causes osteopenia. Endocrinology.

[17] Boers W, Aarrass S, Linthorst C, Pinzani M, Elferink RO, Bosma P (2006). Transcriptional profiling reveals novel markers of liver fibrogenesis: gremlin and insulin-like growth factor-binding proteins. J Biol Chem.

[18] Koli K, Myllärniemi M, Vuorinen K, Salmenkivi K, Ryynänen MJ, Kinnula VL, Keski-Oja J (2006). Bone morphogenetic protein-4 inhibitor gremlin is overexpressed in idiopathic pulmonary fibrosis. Am J Pathol.

[19] Walsh DW, Roxburgh SA, McGettigan P, Berthier CC, Higgins DG, Kretzler M, Cohen CD, Mezzano S, Brazil DP, Martin F (2008). Co-regulation of Gremlin and Notch signalling in diabetic nephropathy. Biochim Biophys Acta.

[20] Corsini M, Moroni E, Ravelli C, Andres G, Grillo E, Ali IH, Brazil DP, Presta M, Mitola S (2014). Cyclic adenosine monophosphate-response element-binding protein mediates the proangiogenic or proinflammatory activity of gremlin. Arterioscler Thromb Vasc Biol.

[21] Mitola S, Ravelli C, Moroni E, Salvi V, Leali D, Ballmer-Hofer K, Zammataro L, Presta M (2010). Gremlin is a novel agonist of the major proangiogenic receptor VEGFR2. Blood.

[22] Yan K, Wu Q, Yan DH, Lee CH, Rahim N, Tritschler I, DeVecchio J, Kalady MF, Hjelmeland AB, Rich JN (2014). Glioma cancer stem cells secrete Gremlin1 to promote their maintenance within the tumor hierarchy. Genes Dev.

[23] Sneddon JB, Zhen HH, Montgomery K, van de Rijn M, Tward AD, West R, Gladstone H, Chang HY, Morganroth GS, Oro AE (2006). Bone morphogenetic protein antagonist gremlin 1 is widely expressed by cancer-associated stromal cells and can promote tumor cell proliferation. Proc Natl Acad Sci U S A.

[24] Kim M, Yoon S, Lee S, Ha SA, Kim HK, Kim JW, Chung J (2012). Gremlin-1 induces BMP-independent tumor cell proliferation, migration, and invasion. PLoS One.

[25] Jaeger E, Leedham S, Lewis A, Segditsas S, Becker M, Cuadrado PR, Davis H, Kaur K, Heinimann K, Howarth K (2012). Hereditary mixed polyposis syndrome is caused by a 40-kb upstream duplication that leads to increased and ectopic expression of the BMP antagonist GREM1. Nat Genet.

[26] Davis H, Irshad S, Bansal M, Rafferty H, Boitsova T, Bardella C, Jaeger E, Lewis A, Freeman-Mills L, Giner FC (2015). Aberrant epithelial GREM1 expression initiates colonic tumorigenesis from cells outside the stem cell niche. Nat Med.

[27] Karagiannis GS, Berk A, Dimitromanolakis A, Diamandis EP (2013). Enrichment map profiling of the cancer invasion front suggests regulation of colorectal cancer progression by the bone morphogenetic protein antagonist, gremlin-1. Mol Oncol.

[28] Ma XJ, Dahiya S, Richardson E, Erlander M, Sgroi DC (2009). Gene expression profiling of the tumor microenvironment during breast cancer progression. Breast Cancer Res.

[29] Kim HS, Shin MS, Cheon MS, Kim JW, Lee C, Kim WH, Kim YS, Jang BG (2017). GREM1 is expressed in the cancer-associated myofibroblasts of basal cell carcinomas. PLoS One.

[30] Wang Y, Klijn JG, Zhang Y, Sieuwerts AM, Look MP, Yang F, Talantov D, Timmermans M, Meijer-van Gelder ME, Yu J (2005). Gene-expression profiles to predict distant metastasis of lymph-node-negative primary breast cancer. Lancet.

[31] Minn AJ, Gupta GP, Padua D, Bos P, Nguyen DX, Nuyten D, Kreike B, Zhang Y, Wang Y, Ishwaran H (2007). Lung metastasis genes couple breast tumor size and metastatic spread. Proc Natl Acad Sci U S A.

[32] Sotiriou C, Wirapati P, Loi S, Harris A, Fox S, Smeds J, Nordgren H, Farmer P, Praz V, Haibe-Kains B (2006). Gene expression profiling in breast cancer: understanding the molecular basis of histologic grade to improve prognosis. J Natl Cancer Inst.

[33] Desmedt C, Piette F, Loi S, Wang Y, Lallemand F, Haibe-Kains B, Viale G, Delorenzi M, Zhang Y, d’Assignies MS (2007). Strong time dependence of the 76-gene prognostic signature for node-negative breast cancer patients in the TRANSBIG multicenter independent validation series. Clin Cancer Res.

[34] Schmidt M, Böhm D, Von Törne C, Steiner E, Puhl A, Pilch H, Lehr H-A, Hengstler JG, Kölbl H, Gehrmann M (2008). The humoral immune system has a key prognostic impact in node-negative breast cancer. Cancer Res.

[35] Riaz M, van Jaarsveld MT, Hollestelle A, Prager-van der Smissen WJ, Heine AA, Boersma AW, Liu J, Helmijr J, Ozturk B, Smid M (2013). miRNA expression profiling of 51 human breast cancer cell lines reveals subtype and driver mutation-specific miRNAs. Breast Cancer Res.

[36] Calon A, Espinet E, Palomo-Ponce S, Tauriello DV, Iglesias M, Céspedes MV, Sevillano M, Nadal C, Jung P, Zhang XH-F (2012). Dependency of colorectal cancer on a TGF-β-driven program in stromal cells for metastasis initiation. Cancer Cell.

[37] McCall MN, Bolstad BM, Irizarry RA (2010). Frozen robust multiarray analysis (fRMA). Biostatistics.

[38] Johnson WE, Li C, Rabinovic A (2007). Adjusting batch effects in microarray expression data using empirical Bayes methods. Biostatistics.

[39] Martens JW, Sieuwerts AM, Bolt-deVries J, Bosma PT, Swiggers SJ, Klijn JG, Foekens JA (2003). Aging of stromal-derived human breast fibroblasts might contribute to breast cancer progression. Thromb Haemost.

[40] Ramkisoensing AA, Pijnappels DA, Askar SF, Passier R, Swildens J, Goumans MJ, Schutte CI, de Vries AA, Scherjon S, Mummery CL (2011). Human embryonic and fetal mesenchymal stem cells differentiate toward three different cardiac lineages in contrast to their adult counterparts. PLoS One.

[41] Ren J, Liu S, Cui C, ten Dijke P (2017). Invasive behavior of human breast cancer cells in embryonic zebrafish. J Vis Exp.

[42] Dennler S, Itoh S, Vivien D, ten Dijke P, Huet S, Gauthier JM (1998). Direct binding of Smad3 and Smad4 to critical TGFβ-inducible elements in the promoter of human plasminogen activator inhibitor-type 1 gene. EMBO J.

[43] Persson U, Izumi H, Souchelnytskyi S, Itoh S, Grimsby S, Engström U, Heldin C-H, Funa K, ten Dijke P (1998). The L45 loop in type I receptors for TGF-β family members is a critical determinant in specifying Smad isoform activation. FEBS Lett.

[44] Krause C, Kloen P, ten Dijke P (2011). Elevated transforming growth factor beta and mitogen-activated protein kinase pathways mediate fibrotic traits of Dupuytren’s disease fibroblasts. Fibrogenesis Tissue Repair.

[45] Naber HP, Wiercinska E, ten Dijke P, van Laar T (2011). Spheroid assay to measure TGF-beta-induced invasion. J Vis Exp.

[46] Buijs J, Van Der Horst G, Van Den Hoogen C, Cheung H, De Rooij B, Kroon J, Petersen M, Van Overveld P, Pelger R, Van Der Pluijm G (2012). The BMP2/7 heterodimer inhibits the human breast cancer stem cell subpopulation and bone metastases formation. Oncogene.

[47] Vogt J, Traynor R, Sapkota GP (2011). The specificities of small molecule inhibitors of the TGFβ and BMP pathways. Cell Signal.

[48] Karnoub AE, Dash AB, Vo AP, Sullivan A, Brooks MW, Bell GW, Richardson AL, Polyak K, Tubo R, Weinberg RA (2007). Mesenchymal stem cells within tumour stroma promote breast cancer metastasis. Nature.

[49] Chen MH, Yeh YC, Shyr YM, Jan YH, Chao Y, Li C-P, Wang SE, Tzeng CH, Chang PMH, Liu CY (2013). Expression of gremlin 1 correlates with increased angiogenesis and progression-free survival in patients with pancreatic neuroendocrine tumors. J Gastroenterol.

[50] Jang BG, Kim HS, Chang WY, Bae JM, Oh HJ, Wen X, Jeong S, Cho NY, Kim WH, Kang GH (2017). Prognostic significance of stromal GREM1 expression in colorectal cancer. Hum Pathol.

[51] Sato M, Kawana K, Fujimoto A, Yoshida M, Nakamura H, Nishida H, Inoue T, Taguchi A, Takahashi J, Adachi K (2016). Clinical significance of Gremlin 1 in cervical cancer and its effects on cancer stem cell maintenance. Oncol Rep.

[52] Grivennikov Sergei I., Greten Florian R., Karin Michael (2010). Immunity, Inflammation, and Cancer. Cell.

[53] Hawinkels LJ, Ten Dijke P (2011). Exploring anti-TGF-β therapies in cancer and fibrosis. Growth Factors.

[54] Guan Y, Cheng W, Zou C, Wang T, Cao Z (2017). Gremlin1 promotes carcinogenesis of glioma in vitro. Clin Exp Pharmacol Physiol.

[55] Yin M, Tissari M, Tamminen J, Ylivinkka I, Ronty M, von Nandelstadh P, Lehti K, Hyytiainen M, Myllarniemi M, Koli K (2017). Gremlin-1 is a key regulator of the invasive cell phenotype in mesothelioma. Oncotarget.

[56] Ravelli C, Mitola S, Corsini M, Presta MJA (2013). Involvement of αvβ3 integrin in gremlin-induced angiogenesis. Angiogenesis.

[57] Lavoz C, Alique M, Rodrigues-Diez R, Pato J, Keri G, Mezzano S, Egido J, Ruiz-Ortega M (2015). Gremlin regulates renal inflammation via the vascular endothelial growth factor receptor 2 pathway. J Pathol.

[58] Ciuclan L, Sheppard K, Dong L, Sutton D, Duggan N, Hussey M, Simmons J, Morrell NW, Jarai G, Edwards M (2013). Treatment with anti–gremlin 1 antibody ameliorates chronic hypoxia/SU5416–induced pulmonary arterial hypertension in mice. Am J Pathol.

[59] Song Kening, Krause Carola, Shi Songting, Patterson Marilyn, Suto Robert, Grgurevic Lovorka, Vukicevic Slobodan, van Dinther Maarten, Falb Dean, ten Dijke Peter, Alaoui-Ismaili Moulay Hicham (2010). Identification of a Key Residue Mediating Bone Morphogenetic Protein (BMP)-6 Resistance to Noggin Inhibition Allows for Engineered BMPs with Superior Agonist Activity. Journal of Biological Chemistry.

[60] Sugimoto H, LeBleu VS, Bosukonda D, Keck P, Taduri G, Bechtel W, Okada H, Carlson W, Bey P, Rusckowski M (2012). Activin-like kinase 3 is important for kidney regeneration and reversal of fibrosis. Nat Med.

[61] Shin K, Lim A, Zhao C, Sahoo D, Pan Y, Spiekerkoetter E, Liao JC, Beachy PA (2014). Hedgehog signaling restrains bladder cancer progression by eliciting stromal production of urothelial differentiation factors. Cancer Cell.

