# Supplementary material for: Cancer-associated fibroblast-derived Gremlin 1 promotes breast cancer progression
Source: Breast Cancer Res. 2019 Sep 18;21:109. doi: 10.1186/s13058-019-1194-0 (PMC6751614; doi:10.1186/s13058-019-1194-0)
Supplement: Supplementary file 2 — Figure S1. Related to Fig. 1. a Kaplan-Meier analysis of metastasis free survival based on GREM1 expression. Endpoint is distant metastasis free survival (MFS). b-e Kaplan-Meier survival analysis of different breast cancer molecular subtypes, HER2+ (b), Triple- (c), ER+ (d), and ER- (e). The subjects were divided into 3 quantiles. Endpoint is distant MFS. f Scatterplot showing the positive correlation between GREM1 and stromal genes / desmoplastic markers expression in clinical datasets. Pearson’s coefficient tests were performed to assess statistical significance. (DOCX 766 kb) [file 13058_2019_1194_MOESM2_ESM.docx]

**Figure S1**. Related to Fig. 1. **a** Kaplan-Meier analysis of metastasis free survival based on *GREM1* expression. Endpoint is distant metastasis free survival (MFS). **b-e** Kaplan-Meier survival analysis of different breast cancer molecular subtypes, HER2^+^ (**b**), Triple^-^ (**c**), ER^+^ (**d**), and ER^-^ (**e**). The subjects were divided into 3 quantiles. Endpoint is distant MFS. **f** Scatterplot showing the positive correlation between *GREM1* and stromal genes / desmoplastic markers expression in clinical datasets. Pearson’s coefficient tests were performed to assess statistical significance.

**Figure S1
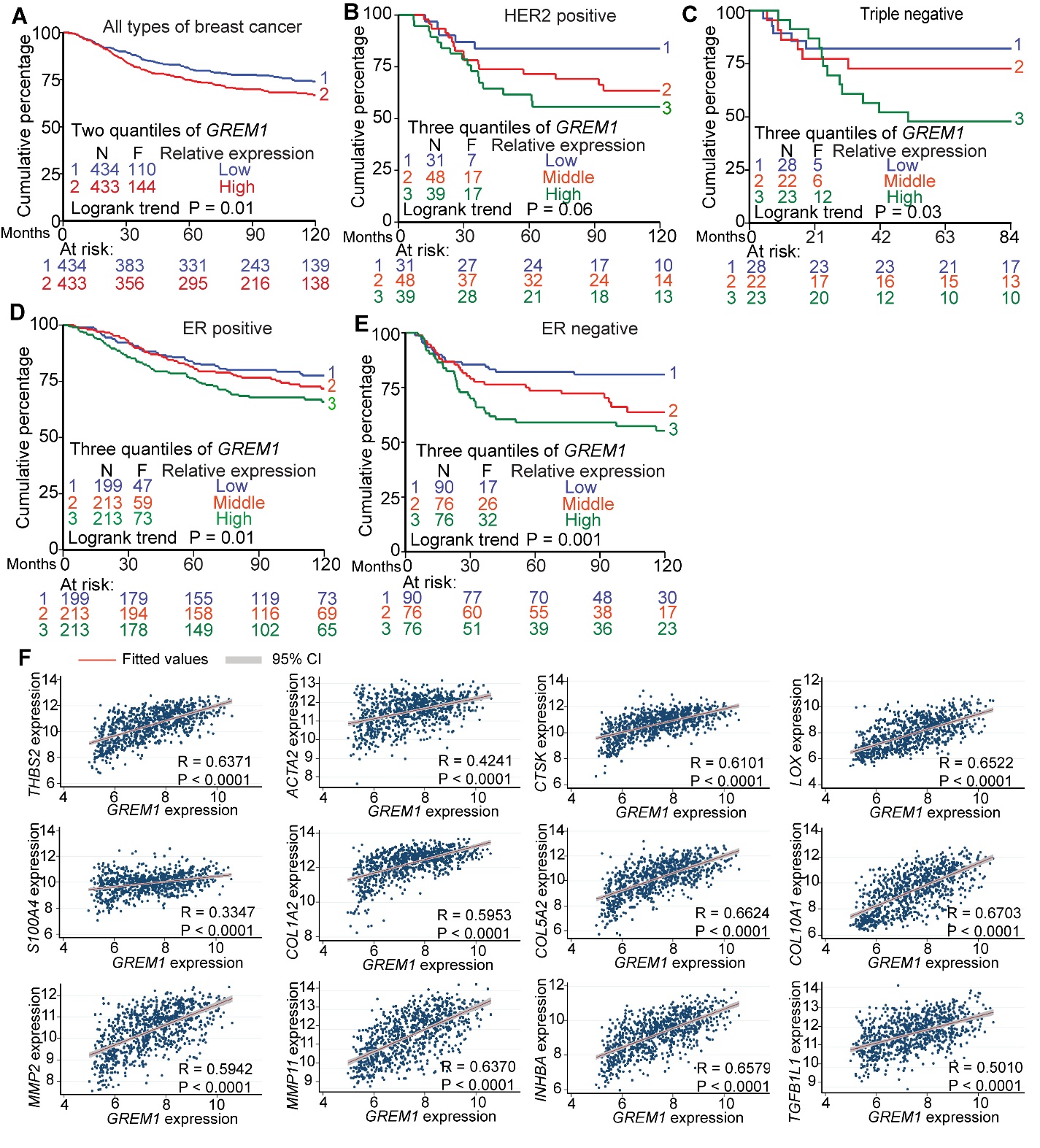
**
