# Supplementary material for: Cancer-associated fibroblast-derived Gremlin 1 promotes breast cancer progression
Source: Breast Cancer Res. 2019 Sep 18;21:109. doi: 10.1186/s13058-019-1194-0 (PMC6751614; doi:10.1186/s13058-019-1194-0)

**Figure S2.** Related to Fig. 2. **a** *GREM1* expression in W21 MSCs after treatment with conditioned medium (CM) from breast cell lines (M1, MDA-MB-21, MCF7). Expression was normalized to the parallel time control of normal medium treatment. The results are expressed as the mean  ±  s.d, n = 3. Student’s t test, **P* $<$ 0.05, ****P* $\leq$ 0.001. **b** TGFβ3 (5 ng/ml), or TNFα (10 ng/ml), or IL1β (10 ng/ml) induces *GREM1* expression in W21 mesenchymal stem cells (MSCs). Expression was normalized to the parallel time control of buffer treatment. The results are expressed as the mean  ±  s.d., n = 3. Student’s t test, **P* $<$ 0.05, ***P* $\leq$ 0.01, ****P* $\leq$ 0.001.

**Figure S2**


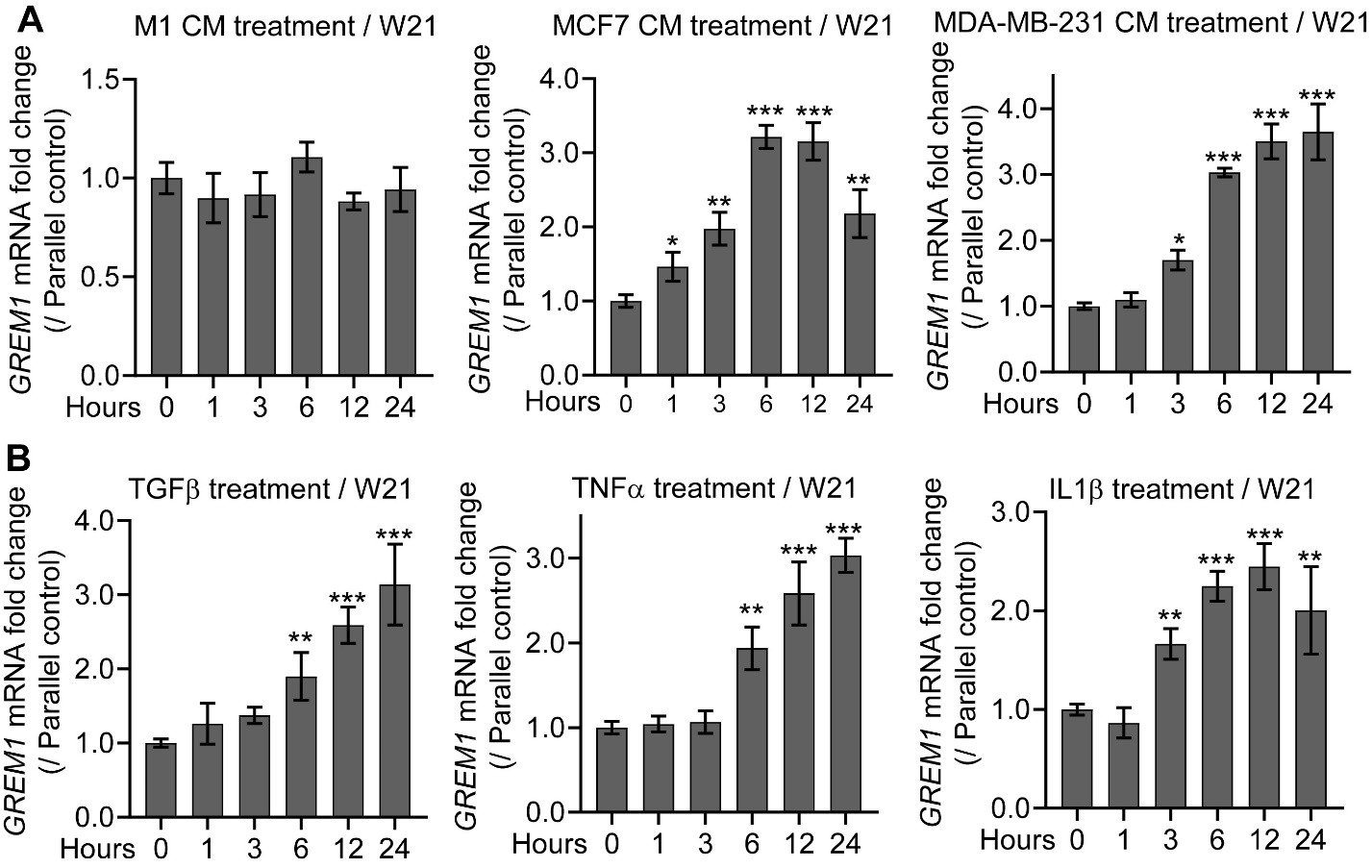

Supplement: Supplementary file 3 — Figure S2. Related to Fig. 2. a GREM1 expression in W21 MSCs after treatment with conditioned medium (CM) from breast cell lines (M1, MDA-MB-21, MCF7). Expression was normalized to the parallel time control of normal medium treatment. The results are expressed as the mean ± s.d, n = 3. Student’s t test, *P < 0.05, ***P ≤ 0.001. b TGFβ3 (5 ng/ml), or TNFα (10 ng/ml), or IL1β (10 ng/ml) induces GREM1 expression in W21 mesenchymal stem cells (MSCs). Expression was normalized to the parallel time control of buffer treatment. The results are expressed as the mean ± s.d., n = 3. Student’s t test, *P < 0.05, **P ≤ 0.01, ***P ≤ 0.001. (DOCX 296 kb) [file 13058_2019_1194_MOESM3_ESM.docx]
