# Supplementary material for: Cancer-associated fibroblast-derived Gremlin 1 promotes breast cancer progression
Source: Breast Cancer Res. 2019 Sep 18;21:109. doi: 10.1186/s13058-019-1194-0 (PMC6751614; doi:10.1186/s13058-019-1194-0)

**Figure S3** Related to Fig. 3. **a**, **b** qRT-PCR measurement for BMPs and BMP receptors in M1, MDA-MB-231 and MCF7 cell lines. ∆Ct values are labeled to show expression abundance. **c** rhGrem1 upregulates stem cell transcription factors in M1 cells. *GAPDH* was used as an internal control. The results are expressed as the mean  ±  s.d., n = 3. Student’s t test, **P* $<$ 0.05, ***P* $\leq$ 0.01.

**Figure S3**


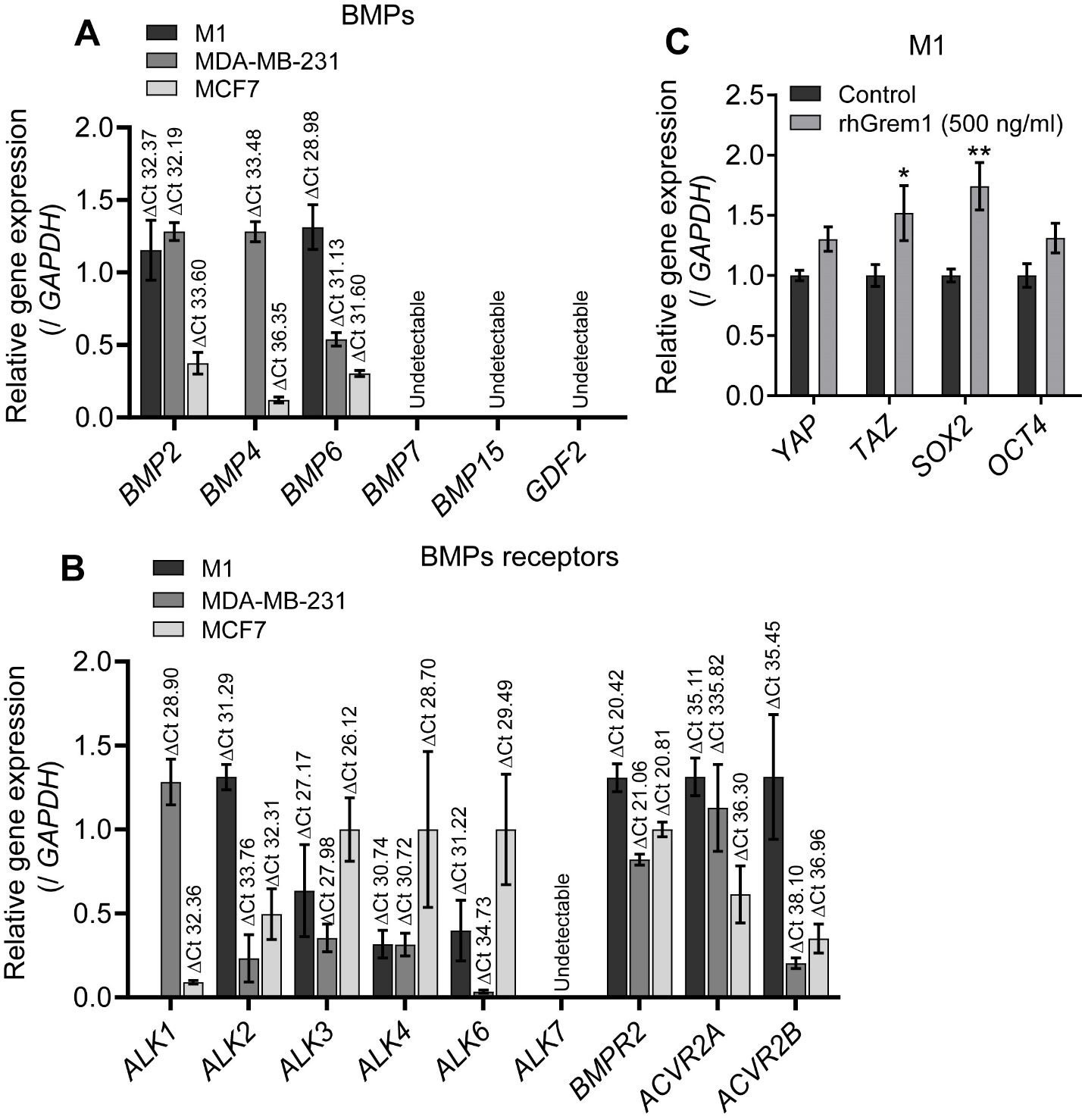

Supplement: Supplementary file 4 — Figure S3. Related to Fig. 3. a, b qRT-PCR measurement for BMPs and BMP receptors in M1, MDA-MB-231 and MCF7 cell lines. ∆Ct values are labeled to show expression abundance. c rhGrem1 upregulates stem cell transcription factors in M1 cells. GAPDH was used as an internal control. The results are expressed as the mean ± s.d., n = 3. Student’s t test, *P < 0.05, **P ≤ 0.01. (DOCX 368 kb) [file 13058_2019_1194_MOESM4_ESM.docx]
