# Supplementary material for: Cancer-associated fibroblast-derived Gremlin 1 promotes breast cancer progression
Source: Breast Cancer Res. 2019 Sep 18;21:109. doi: 10.1186/s13058-019-1194-0 (PMC6751614; doi:10.1186/s13058-019-1194-0)
Supplement: Supplementary file 5 — Figure S4. Related to Fig. 4. a GREM1 OE upregulates the expression of EMT transcription factors and markers in M1 cells. GAPDH was used as an internal control. The results are expressed as the mean ± s.d., n = 3. Student’s t test, *P < 0.05, **P ≤ 0.01, ***P ≤ 0.001. b exogenous administration of rhGrem1 inhibits BMP-induced SMAD1/5/8 phosphorylation (pSMAD1/5/8) in MDA-MB-231 and M2 cell lines. (DOCX 175 kb) [file 13058_2019_1194_MOESM5_ESM.docx]

**Figure S4** Related to Fig. 4. **a** *GREM1* OE upregulates the expression of EMT transcription factors and markers in M1 cells. *GAPDH* was used as an internal control. The results are expressed as the mean  ±  s.d., n = 3. Student’s t test, **P* $<$ 0.05, ***P* $\leq$ 0.01, ****P* $\leq$ 0.001. **b** exogenous administration of rhGrem1 inhibits BMP-induced SMAD1/5/8 phosphorylation (pSMAD1/5/8) in MDA-MB-231 and M2 cell lines.

**Figure S4
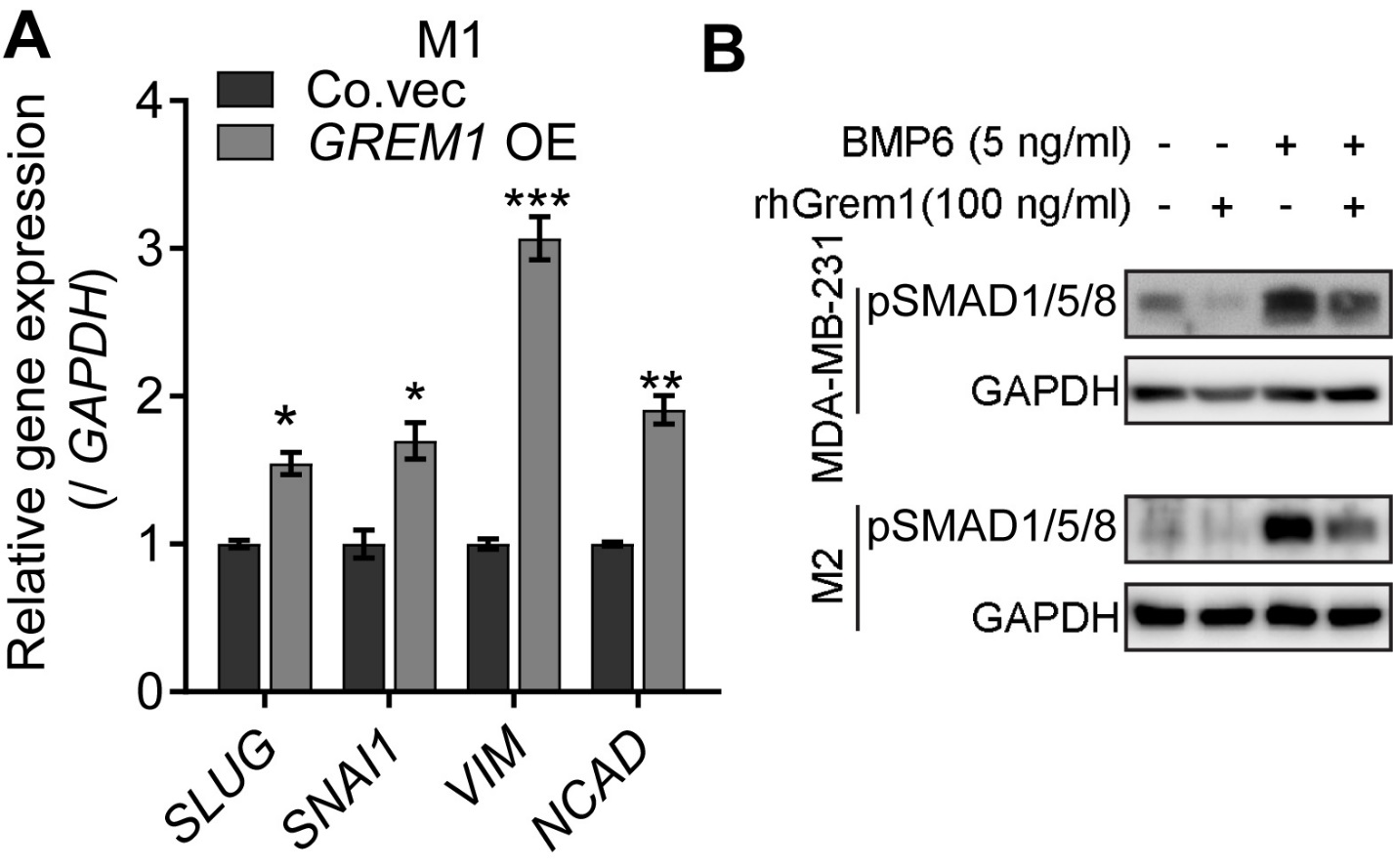
**
