# Supplementary material for: Cancer-associated fibroblast-derived Gremlin 1 promotes breast cancer progression
Source: Breast Cancer Res. 2019 Sep 18;21:109. doi: 10.1186/s13058-019-1194-0 (PMC6751614; doi:10.1186/s13058-019-1194-0)
Supplement: Supplementary file 6 — Figure S5. Related to Fig. 5. GREM1 overexpression (OE) in fetal mesenchymal stem cells (MSCs) W21 shows fibroblast-like characteristics. a Stable GREM1 OE in MSCs W21 inhibits BMP6 (5 ng/ml) induced SMAD1/5/8 phosphorylation (pSMAD1/5/8). Left, relative mRNA level determined by qRT-PCR. GAPDH was used as internal control. The results are expressed as the mean ± s.d., n = 3. Student’s t test, ***P ≤ 0.001. b qRT-PCR analysis of selected BMP targets, TGFβb pathway constituents/targets, fibroblast activation markers, matrix metalloproteinases, in W21 MSCs with/without GREM1 stable OE. GAPDH was used as internal control. The results are expressed as the mean ± s.d., n = 3. Student’s t test, *P < 0.05, **P ≤ 0.01, ***P ≤ 0.001. c Western blot to detect indicated proteins level change after GREM1 OE in W21 MSCs. d W21 MSCs with/without GREM1 OE were stained with fluorescein-phalloidin (green) to visualize F-actin. DAPI was used for nuclear staining (blue). e Collagen gel contraction assay. W21 MSCs with/without GREM1 OE were embedded in collagen gels. After 24, 48, and 72 h, the area of each gel (white dash circle) was imaged and quantified. Left, representative images of contracted gels. Right, percentage of gel contraction gel. Quantification is shown in Methods. The results are expressed as the mean ± s.d., n = 3. Student’s t test, *P < 0.05, **P ≤ 0.01. f qRT-PCR analysis of selected genes in W21 MSCs after 48 hours treatment with recombinant human Grem1 (rhGrem1) protein (500 ng/ml) or BMP type I receptors inhibitor LDN193198 (120 nM). GAPDH was used as internal control. The results are expressed as the mean ± s.d., n = 3. Student’s t test, *P < 0.05, **P ≤ 0.01, ***P ≤ 0.001. (DOCX 447 kb) [file 13058_2019_1194_MOESM6_ESM.docx]

**Figure S5** Related to Fig. 5. *GREM1* overexpression (OE) in fetal mesenchymal stem cells (MSCs) W21 shows fibroblast-like characteristics. **a** Stable *GREM1* OE in MSCs W21 inhibits BMP6 (5 ng/ml) induced SMAD1/5/8 phosphorylation (pSMAD1/5/8). Left, relative mRNA level determined by qRT-PCR. *GAPDH* was used as internal control. The results are expressed as the mean  ±  s.d., n = 3. Student’s t test, ****P* $\leq$ 0.001. **b** qRT-PCR analysis of selected BMP targets, TGF pathway constituents/targets, fibroblasts activation markers, matrix metalloproteinases, in W21 MSCs with/without *GREM1* stable OE. *GAPDH* was used as internal control. The results are expressed as the mean  ±  s.d., n = 3. Student’s t test, **P* $<$ 0.05, ***P* $\leq$ 0.01, ****P* $\leq$ 0.001. **c** Western blot to detect indicated proteins level change after *GREM1* OE in W21 MSCs. **d** W21 MSCs with/without *GREM1* OE were stained with fluorescein-phalloidin (green) to visualize F-actin. DAPI was used for nuclear staining (blue). **e** Collagen gel contraction assay. W21 MSCs with/without *GREM1* OE were embedded in collagen gels. After 24, 48, and 72 h, the area of each gel (white dash circle) was imaged and quantified. Left, representative images of contracted gels. Right, percentage of gel contraction gel. Quantification is shown in Methods. The results are expressed as the mean   ±  s.d., n = 3. Student’s t test, **P* $<$ 0.05, ***P* $\leq$ 0.01. **f** qRT-PCR analysis of selected genes in W21 MSCs after 48 hours treatment with recombinant human Grem1 (rhGrem1) protein (500 ng/ml) or BMP type I receptors inhibitor LDN193198 (120 nM). *GAPDH* was used as internal control. The results are expressed as the mean  ±  s.d., n = 3. Student’s t test, **P* $<$ 0.05, ***P* $\leq$ 0.01, ****P* $\leq$ 0.001.

**Figure S5**

**
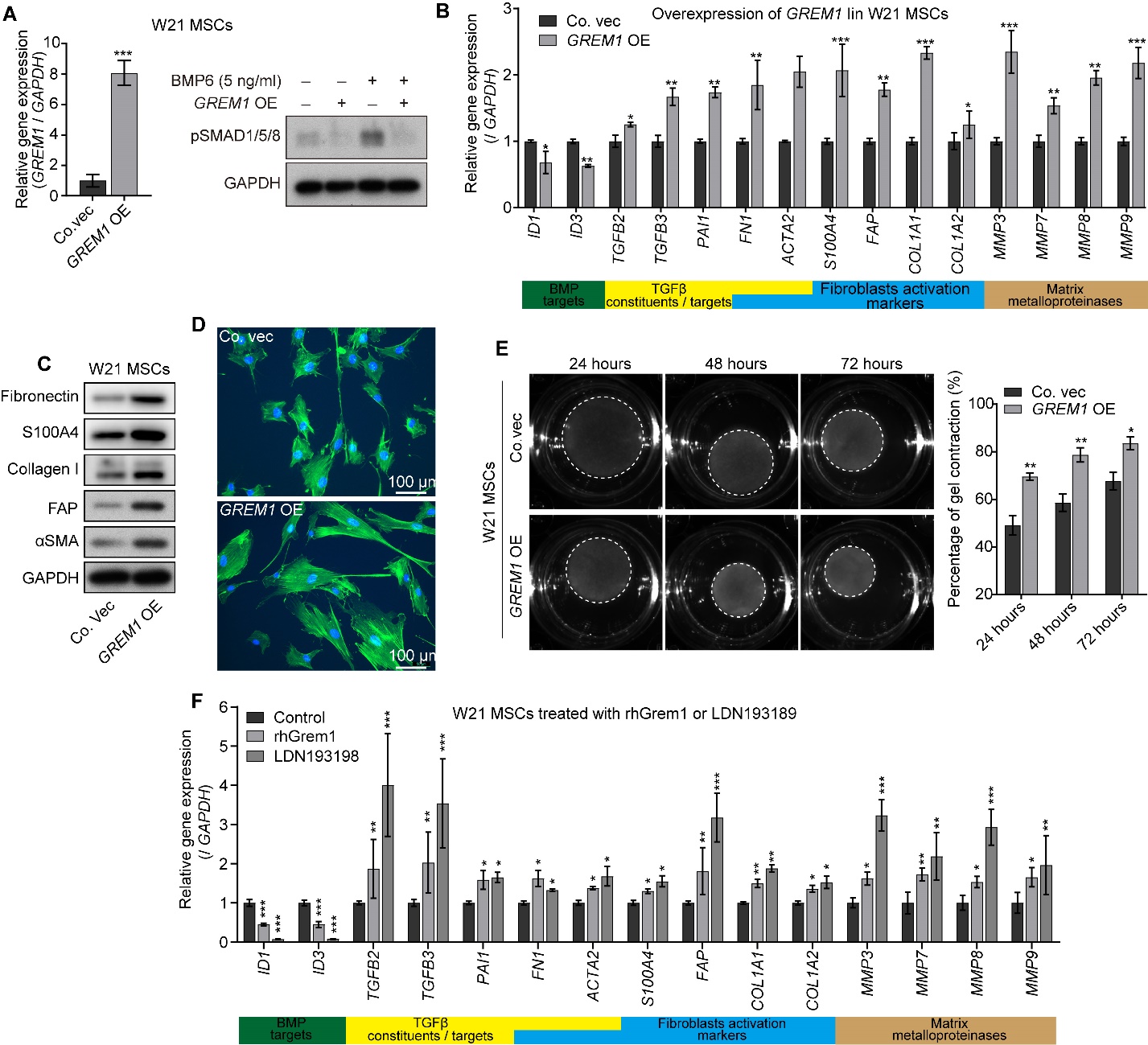
**
