# Supplementary material for: Cancer-associated fibroblast-derived Gremlin 1 promotes breast cancer progression
Source: Breast Cancer Res. 2019 Sep 18;21:109. doi: 10.1186/s13058-019-1194-0 (PMC6751614; doi:10.1186/s13058-019-1194-0)
Supplement: Supplementary file 7 — Figure S6. Related to Fig. 6. Spheroid invasion assays. a Schematic illustration of spheroid production. Briefly, mCherry-labeled MDA-MB-231 or MCF7 cells (Red) were mixed with AmCyan (converted to blue)-labeled 19TT breast cancer-associated fibroblasts (CAFs) at a ratio of 1:1. Mixtures were cultured for 7 days in hanging drops to obtain spheroids. b 19TT CAFs promotes MCF7 cells invasion. Left, representative images of spheroids at days 0, 2, and 4. Red, MCF7 cells; Blue, 19TT CAFs. Right, the relative invasion area was quantified as area difference at days 2 and 4, relative to day 0. The results are expressed as the as the mean ± s.d., n = 8. Student’s t test, **P ≤ 0.01. (DOCX 197 kb) [file 13058_2019_1194_MOESM7_ESM.docx]

**Figure S6.** Related to Fig. 6. Spheroid invasion assays. **a** Schematic illustration of spheroid production. Briefly, mCherry-labeled MDA-MB-231 or MCF7 cells (Red) were mixed with AmCyan (converted to blue)-labeled 19TT breast cancer-associated fibroblasts (CAFs) at a ratio of 1:1. Mixtures were cultured for 7 days in hanging drops to obtain spheroids. **b** 19TT CAFs promotes MCF7 cells invasion. Left, representative images of spheroids at days 0, 2, and 4. Red, MCF7 cells; Blue, 19TT CAFs. Right, the relative invasion area was quantified as area difference at days 2 and 4, relative to day 0. The results are expressed as the as the mean   ±  s.d., n = 8. Student’s t test, ***P* $\leq$ 0.01.

**Figure S6**

**
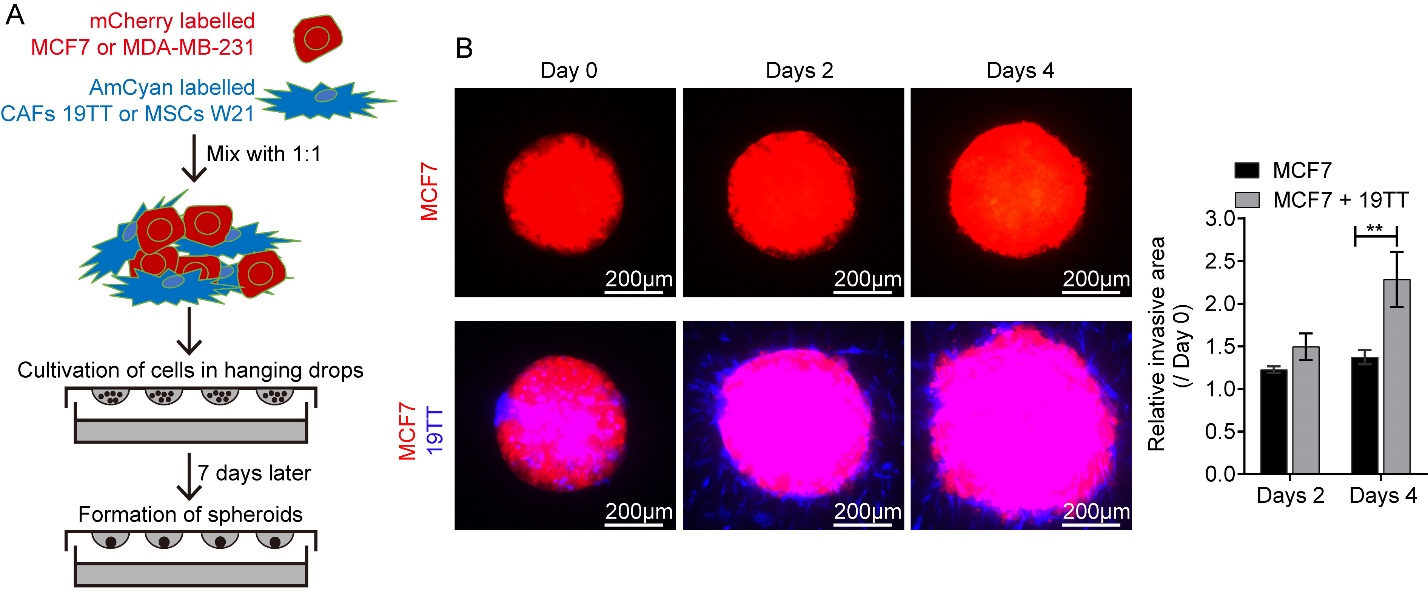
**
