# Supplementary material for: Cancer-associated fibroblast-derived Gremlin 1 promotes breast cancer progression
Source: Breast Cancer Res. 2019 Sep 18;21:109. doi: 10.1186/s13058-019-1194-0 (PMC6751614; doi:10.1186/s13058-019-1194-0)
Supplement: Supplementary file 8 — Figure S7. Related to Fig. 7. GREM1 overexpression (OE) in W21 mesenchymal stem cells (MSCs) promotes breast cancer cells intravasation in zebrafish embryo perivitelline space coinjection model. Perivitelline space co-injection of MDA-MB-231 cells and W21 MSCs with/without GREM1 stable OE. The panels show representative images. Green, endothelium of zebrafish; Red, mCherry-labelled MDA-MB-231; Blue, converted from AmCyan-labelled W21. Yellow arrowheads point to single intravasated cells in the head and tail regions of zebrafish. Left, cells migration in the perivitelline space; middle, image of zebrafish embryo body; Right, visualization of intravasated cells in the posterior of embryo. The graph shows quantification of the number of intravasated cells in each embryonic body at 3 days post injection (dpi). The results are expressed as the mean ± s.e.m., n=2. Student’s t test, **P ≤ 0.01. (DOCX 269 kb) [file 13058_2019_1194_MOESM8_ESM.docx]

**Figure S7**. Related to Fig. 7. *GREM1* overexpression (OE) in W21 mesenchymal stem cells (MSCs) promotes breast cancer cells intravasation in zebrafish embryo perivitelline space coinjection model. Perivitelline space co-injection of MDA-MB-231 cells and W21 MSCs with/without *GREM1* stable OE. The panels show representative images. Green, endothelium of zebrafish; Red, mCherry-labelled MDA-MB-231; Blue, converted from AmCyan-labelled W21. Yellow arrowheads point to single intravasated cells in the head and tail regions of zebrafish. Left, cells migration in the perivitelline space; middle, image of zebrafish embryo body; Right, visualization of intravasated cells in the posterior of embryo. The graph shows quantification of the number of intravasated cells in each embryonic body at 3 days post injection (dpi). The results are expressed as the mean  ±  s.e.m., n=2. Student’s t test, ***P* $\leq$ 0.01.

**Figure S7**

**
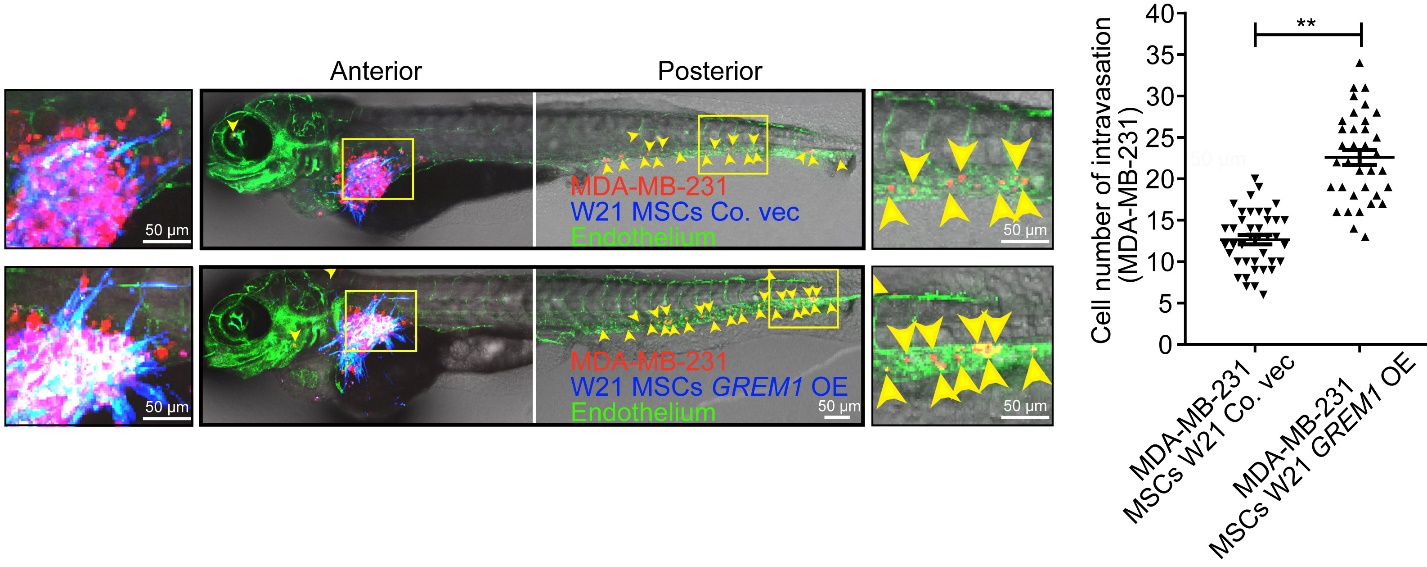
**
