# Supplementary material for: Cancer-associated fibroblast-derived Gremlin 1 promotes breast cancer progression
Source: Breast Cancer Res. 2019 Sep 18;21:109. doi: 10.1186/s13058-019-1194-0 (PMC6751614; doi:10.1186/s13058-019-1194-0)

**Figure S8.** Related to Fig. 1. **a** *GREM1* mRNA expression in epithelium and stroma compartments in breast cancer dataset GSE14548. Epithelium and stroma were extracted from normal breast, grade I, II, III ductal carcinoma *in situ* (DCIS) and invasive breast cancer tissue using laser capture. **b** *GREM1* expression in epithelial cells, leukocytes, fibroblasts and endothelial cells in colorectal cancer dataset GSE39396. The each specific type of cells were isolated by flow cytometry.

**Figure S8**


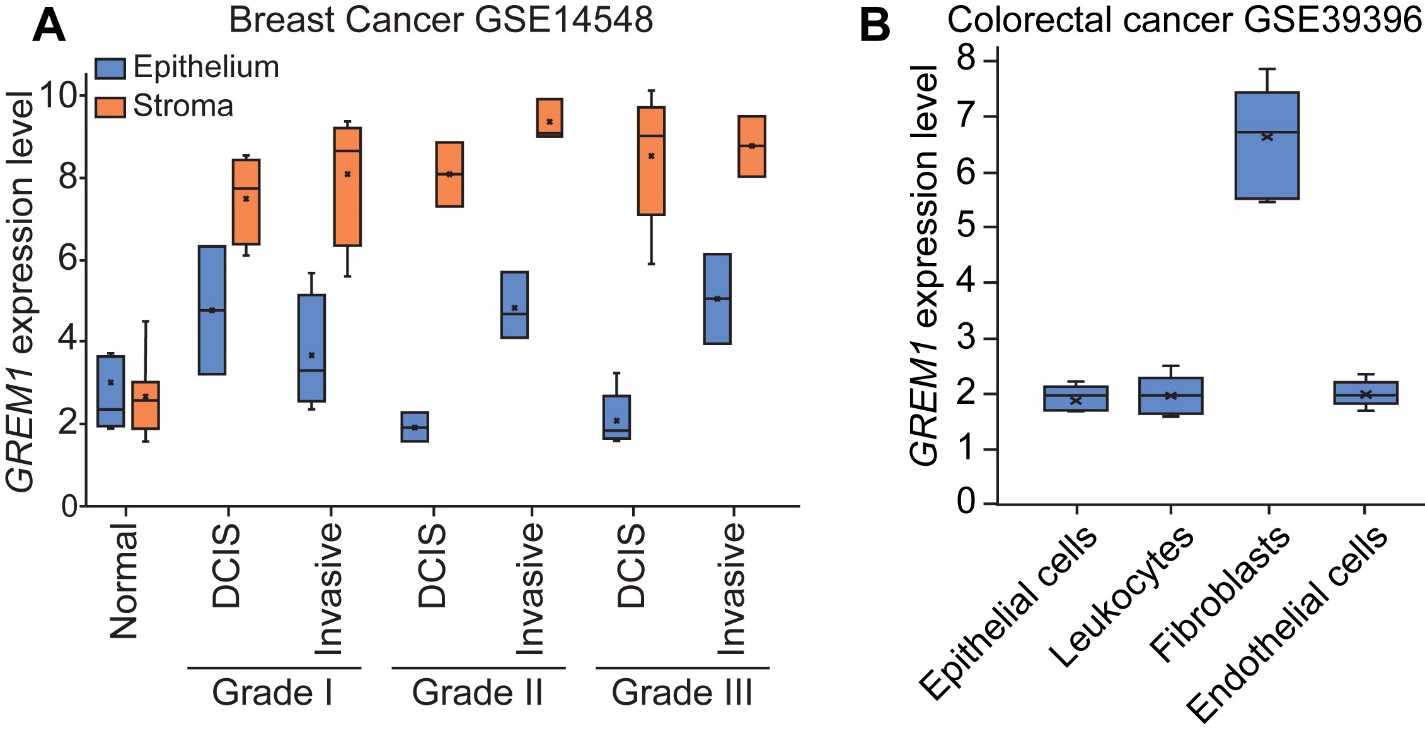

Supplement: Supplementary file 9 — Figure S8. Related to Fig. 1. a GREM1 mRNA expression in epithelium and stroma compartments in breast cancer dataset GSE14548. Epithelium and stroma were extracted from normal breast, grade I, II, III ductal carcinoma in situ (DCIS) and invasive breast cancer tissue using laser capture. b GREM1 expression in epithelial cells, leukocytes, fibroblasts and endothelial cells in colorectal cancer dataset GSE39396. The each specific type of cells were isolated by flow cytometry. (DOCX 177 kb) [file 13058_2019_1194_MOESM9_ESM.docx]
